# A General Strategy for Bandgap Engineering Via Anion‐Lattice Doping in High‐Entropy Oxides

**DOI:** 10.1002/advs.202505789

**Published:** 2025-06-19

**Authors:** Kevin Siniard, Juntian Fan, Meijia Li, Qingju Wang, Alexander S. Ivanov, Tao Wang, Sheng Dai

**Affiliations:** ^1^ Department of Chemistry Institute for Advanced Materials and Manufacturing University of Tennessee Knoxville Knoxville TN 37996 USA; ^2^ Chemical Sciences Division Oak Ridge National Laboratory Oak Ridge TN 37831 USA

**Keywords:** energy storage, high entropy, mechanochemistry, solid solution

## Abstract

Bandgap engineering is a critical tool for tailoring the electronic properties of functional materials, traditionally achieved by modifying the cation sublattice. Here, a generalizable strategy is introduced that leverages facile anion‐lattice doping in high entropy materials to modulate the bandgap in high‐entropy metal oxides (HEMOs). By incorporating nitrogen into a single‐phase high‐entropy metal oxide/nitride (HEMO:HEMN) solid solution, a substantial bandgap reduction is achieved from 3.55 eV (HEMO) to ≈2.46 eV (HEMO:HEMN), significantly enhancing electronic conductivity. Unlike conventional bandgap tuning approaches that rely on cation substitution or heterojunction formation, this method exploits anion‐mediated entropy stabilization, enabling uniform bandgap narrowing across the entire solid solution. This anion‐lattice engineering strategy is broadly applicable to high‐entropy systems, providing a new pathway for designing energy materials with tailored electronic properties. The resulting HEMO:HEMN solid solution exhibits a tenfold increase in capacitance and capacity compared to HEMO in supercapacitor and lithium‐ion battery tests, demonstrating the transformative potential of anion‐driven bandgap modulation for next‐generation energy storage and conversion technologies.

## Graphical abstract

A generalizable strategy is developed leveraging facile anion‐lattice doping in high entropy materials to modulate the bandgap in high‐entropy metal oxides (HEMOs). By incorporating nitrogen into a single‐phase high‐entropy metal oxide/nitride (HEMO:HEMN) solid solution, a substantial bandgap reduction from 3.55 eV (HEMO) to ≈2.46 eV (HEMO:HEMN) is acheived, significantly enhancing electronic conductivity.

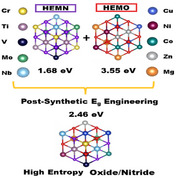

## 1 Introduction

Given the current energy demands, the synthesis of novel electrochemical energy storage materials stands at the forefront of chemical and materials research. The development of supercapacitors and lithium‐ion batteries (LIBs) play an important role in satisfying higher energy demands due to fast charge and discharge capabilities and higher power densities.^[^ ^1^, ^2^, ^3^ ^]^ The most important materials currently utilized in the fabrication of supercapacitors and LIBs include carbon‐based materials, metal oxides, metal nitrides, and conducting polymers.^[^ ^1^, ^2^, ^3^ ^]^ Recently, the development of materials for energy storage devices has extended to high entropy materials (HEMs) where five or more metal cations exist within a single‐phase crystalline structure.^[^ ^1^, ^2^, ^3^ ^]^ To date, there are several high entropy materials that exhibit excellent electrochemical capabilities. Among these include high entropy metal nitrides (HEMNs) (e.g., ZrVNbMoCrN_x_)^[^ ^4^, ^5^ ^]^ and high entropy metal oxides (HEMOs) (e.g., NiMgZnCoCuO_x_),^[^ ^6^, ^7^ ^]^ with each possessing unique physical characteristics, electrochemical behavior, and synthesis routes that lead to enhanced capacitance in supercapacitors and lithium‐ion storage in batteries.^[^ ^7^, ^8^, ^9^ ^]^

The unique electrochemical properties of HEMNs and HEMOs can be understood energetically and physically based on their respective transition metal constituents. For instance, transition metal oxides (TMOs) possess highly reversible redox reactions for potentially large pseudo‐capacitance. Regarding HEMOs, the electrochemical redox reactions that take place within energy storage devices is attributed to the reduction and oxidation of the transition metal oxides (M^+4/+3^ M^+2^ M^0^).^[^ ^10^, ^11^, ^12^, ^13^, ^14^ ^]^ Unfortunately, TMOs commonly suffer from low utilization of active sites due to their low conductivity (e.g., 5.6 × 10^−6^ S cm^−1^ for NiO), a consequence of a large bandgap (>3 eV).^[^ ^15^ ^]^ Thus, a combination with highly conductive materials such as carbon nanotubes (CNTs) or other high entropy metal deposits is often employed to increase conductivity.^[^ ^16^, ^17^ ^]^ For instance, lithium doping in MgNiCoCuZnO has been shown to induce bandgap narrowing from 1.0 to 0.6 eV through a charge compensation mechanism, where the stabilization of transition metals in the ^3+^ oxidation state contributes to electronic structure modifications.^[^ ^16^, ^17^ ^]^ Moreover, variations in electronegativity and crystal field splitting among transition metals in (Fe₀.₂Co₀.₂Ni₀.₂Cu₀.₂Zn₀.₂)Al₂O₄ broaden the 3d states, resulting in a 0.9 eV bandgap reduction compared to the precursor oxides.^[^ ^18^ ^]^

Building upon these cation‐based entropy tuning strategies, the approach presented herein further incorporates anion doping as an additional degree of freedom to achieve a more pronounced bandgap reduction. For instance, HEMNs and their constituent transition metal nitrides (TMNs) demonstrate high capacitance through charge separation that occurs at an electric interface resulting in the creation of an electric double layer (EDL), allowing TMN‐based supercapacitors to deliver high power density and fast charge/discharge rates.^[^ ^19^, ^20^, ^21^, ^22^, ^23^, ^24^, ^25^, ^26^, ^27^ ^]^ The conductivity of TMNs is ten orders of magnitude higher than that of TMOs (e.g., 4 × 10^4^ S cm^−1^ for TiN) due to a more narrow bandgap (< 3 eV).^[^ ^28^ ^]^ However, TMNs generally suffer from low energy density due to limited redox sites.^[^ ^29^, ^30^, ^31^ ^]^ Therefore, materials that demonstrate the combined features of EDL capacitance and pseudo‐capacitor redox reactions are necessary to resolve the low conductivity and energy density issues inherent in TMOs and TMNs.^[^ ^1^, ^32^, ^33^, ^34^ ^]^

Recently, the construction of oxides/nitride heterostructures has proven effective at generating active interfaces that circumvent the insufficiencies of low conductivity and further modify the bandgap of materials.^[^ ^35^, ^36^ ^]^ However, heterojunctions inherently involve multiple phases, leading to interfacial defects, strain, and phase segregation, which can introduce undesirable electronic states that limit charge transport and long‐term stability. Additionally, contemporary methodologies to produce oxide/nitride heterostructures have traditionally leaned upon techniques such as atomic layer deposition (ALD), electrospinning, and high‐temperature processes, including hydrothermal and sol‐gel synthesis. Regrettably, these methods predominantly involve interfacial atoms, thus constricting the effective utilization of the majority of atoms residing within the bulk phase.^[^ ^4^, ^37^ ^]^ While several advancements toward the fabrication of HEMs are evident, the synthesis of a combined high entropy solid solution under facile and ambient conditions remains challenging due to contrasting chemical environments. For instance, the synthesis of HEMOs requires an oxidative atmosphere, while HEMNs require an inert atmosphere, imposing limitations on traditional calcination processes.^[^ ^36^ ^]^ Additionally, achieving a single‐phase solid solution is crucial for reducing the bandgap effectively, as phase segregation in heterojunctions can introduce interfacial states that hinder electronic properties. However, current techniques face challenges due to lattice mismatch, which complicates the formation of a stable single‐phase solid solution. Therefore, developing novel and straightforward approaches to efficiently and controllably construct extensive high entropy solid solutions—capable of further enhancing configurational disorder through both cation and anion doping to achieve modified conductivity through bandgap reductions, particularly under scalable and ambient conditions—remains a long‐standing challenge.

In this work, a facile mechanochemical method is employed to combine a HEMN and a HEMO that circumvents the need for consolidated, paradoxical chemical synthesis environments. The high entropy oxide/nitride solid solutions reported herein feature ten metal cations including (V, Ti, Cr, Mo, Nb, Mn, Fe, Ni, Cu, Zn) that coordinate with O and N anion sites, utilizing a unique entropic combinatory arrangement of two different HEMs in a single‐phase solid solution that is different from the starting materials. This unique anion–cation co‐engineering strategy not only builds upon existing cation‐based high entropy systems but also leverages additional anion‐mediated entropy to further reduce the bandgap, setting this approach apart from conventional heterojunction methods.

## 2 Results and Discussion

### 2.1 Synthesis and Characterization of High Entropy Oxy/Nitride Solid Solution

The distinctive entropic setting of the novel HEMO:HEMN high‐entropy solid solution can be clarified through theoretical entropy calculations.

(1) $$S_{\mathrm{conf}\mathrm{ig}}=-R\left[{\left(\sum_{a=1}^{n}x_{a}\ln x_{a}\right)}_{a-\mathrm{site}}+{\left(\sum_{b=1}^{n}x_{b}\ln x_{b}\right)}_{b-\mathrm{Site}}\right]$$

Notably, the entropy increases as the coordination changes from 5 cations and 1 anion in the starting materials to 10 cations with 2 anion sites resulting in a theoretical entropy of 2.99R (0.024 kJ mol^−1^K^−1^) for the high entropy oxide/nitride solid solution compared to 1.61R (0.0133 kJ mol^−1^K^−1^) for HEMN and HEMO respectively. The formation of HEMO:HEMN 1:1 solid solution reduces the bandgap from 3.55 eV (HEMO) to ≈2.46 eV, significantly enhancing electronic conductivity. The novel oxide/nitride solid solutions presented herein demonstrate excellent electrochemical storage capabilities that outperform the precursor HEMs in both supercapacitor and LIB tests.

**Figure 1:**
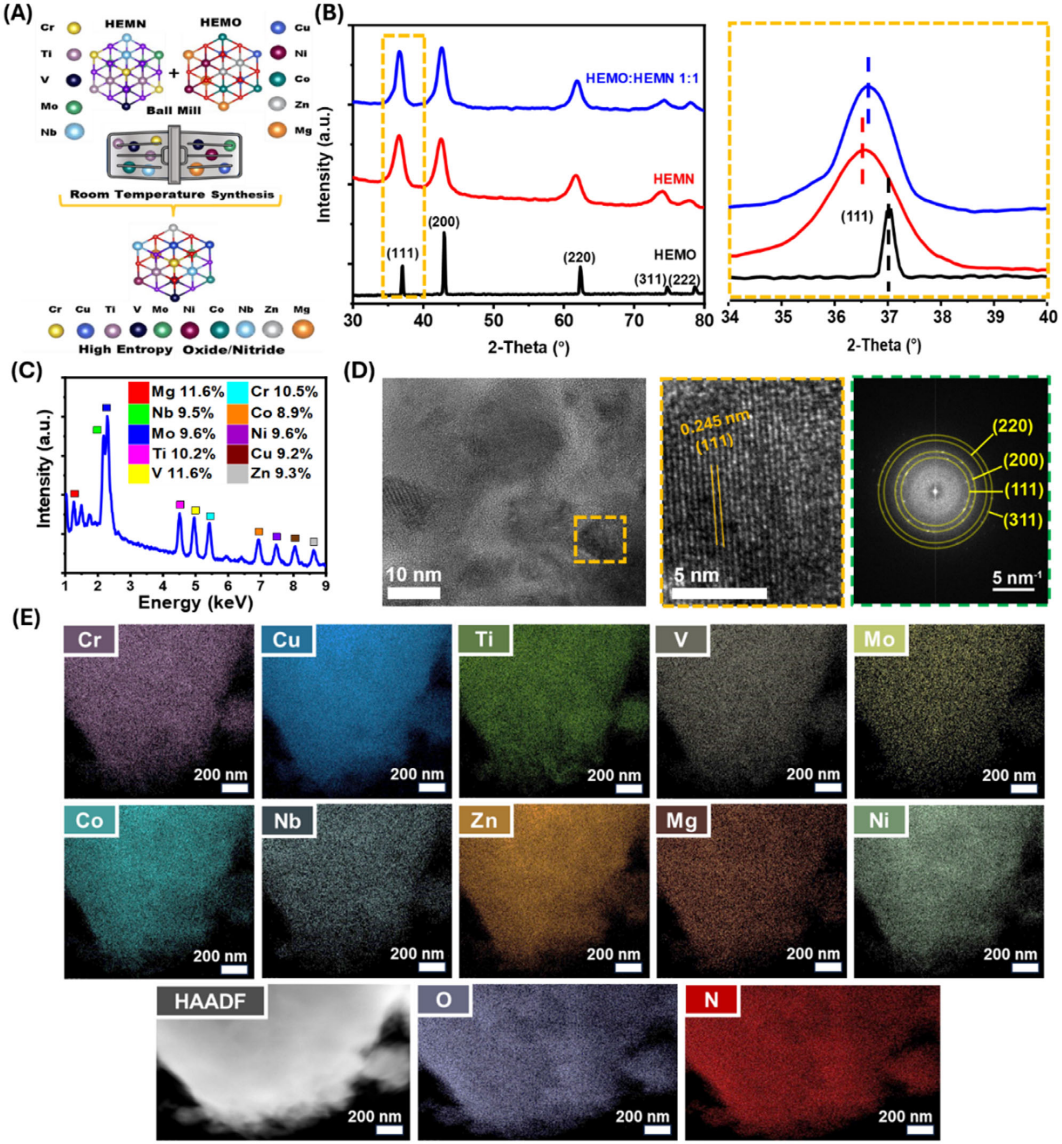
A) Schematic illustration showing the synthesis of the High Entropy Oxide/Nitride Solid Solutions. B) XRD analysis of HEMO, HEMN, HEMO:HEMN 1:1 solid solutions with enlarged area marked in orange. C) Relative atomic metal compositions of HEMO:HEMN 1:1 from EDS. D) HRTEM image of HEMO:HEMN 1:1 with lattice planes and FFT pattern shown in the orange box and green boxes. E) HAADF‐STEM and EDS mapping of HEMO:HEMN 1:1 featuring Cr, Cu, Ti, V, Mo, Co, Nb, Zn, Mg, Ni, O, and N signals.

The HEMN (TiVNbMoCrN_x_) was synthesized according to our previous work by using five metal equimolar salt precursors featuring (V, Ti, Cr, Mo, Nb) via mechanochemical ball‐mill and inert N_2_(g) calcination at 850 °C.^[^ ^4^ ^]^ Additionally, five equimolar metal salt precursors featuring (Mg, Co, Ni, Cu, Zn) were used for the fabrication of the HEMO (NiMgZnCoCuO_x_) via mechanochemical ball‐mill and calcination in air at 900 °C.^[^ ^38^ ^]^ The resulting HEMs were then ball milled together in a select molar ratios of 1:1 (HEMO:HEMN 1:1) or 2:1 (HEMO:HEMN 2:1) yielding the high entropy oxide/nitride single‐phase solid solution as illustrated in **Figure** **1**A.

X‐ray Diffraction (XRD) (Figure 1B) analysis confirms the successful combination of the mechanochemically synthesized HEMs into a single‐phase solid solution with each select ratioed oxide/nitride solid solution exhibiting five broad peaks that correspond to the (111), (200), (220), (311), and (222) planes of a cubic F*m*3̅*m* rock‐salt phase. The successful combination of the lattices from each HEM is established by the observed (111) peak shift from 37.05° to 36.65° for the HEMO and from 35.55° to 36.65° for the HEMN to HEMO:HEMN 1:1. The observed XRD peak shifts are outlined in the orange box region corresponding to Figure 1B. Rietveld refinement was performed for the XRD pattern of HEMN:HEMO 1:1 to identify any potential secondary phases. As shown in Figure S1 (Supporting Information), the calculated data fits the experimental data very well, using a cubic Fm3̅m rock‐salt phase. The weighted profile residual (R_wp_) is only 2.23%, indicating the single‐phase formation of HEMN:HEMO 1:1. Additionally, the XRD pattern of HEMO:HEMN 2:1 compared to the starting HEMs is showcased in Figure S2 (Supporting Information). The entropic contributions that lead toward formation of novel solid solution HEMs can be further understood according to the Gibbs free energy, entropy forming ability (EFA), and Boltzmann's entropy formula.^[^ ^4^, ^39^, ^40^, ^41^ ^]^

(2) ΔG=ΔH−TΔS

(3) $$EFA={\left[\left(\frac{\sum_{i=1}^{n}g_{i}{\left(H_{i}-H\right)}^{2}}{\left(\sum_{i=1}^{n}g_{i}\right)-1}\right)\right]}^{-1}$$

(4) $$S_{c\;}=k_{B}\log\left(\Omega \right)$$

Based on Gibbs Free energy evaluations, competing entropic and enthalpic effects lead toward free energy minimization and high temperature stability.^[^ ^4^, ^39^, ^40^, ^41^ ^]^ Furthermore, according to calculated EFA values, entropic effects are among the main factors in producing a single‐phase solid solution. The EFA is defined as the inverse of the standard deviation of the energy distribution spectrums of all possible cation configurations.^[^ ^9^, ^41^ ^]^ Thus, corresponding EFA values have implications on single‐phase solid solution formability.^[^ ^41^ ^]^ Accordingly, owing to their low enthalpic formation and high entropic contributions, both HEMO and HEMN have high EFA values alluding to their favorable formation toward a high entropy solid solution.^[^ ^9^ ^]^ Additionally, the increase in entropy can be understood according to Boltzmann's entropy formula, where the involvement of the several metal sites that exist within each HEM increases the number of possible random configurations thus increasing the configurational entropy of the system. Therefore, the combination of HEMO and HEMN toward the generation of a novel high entropy oxide/nitride solid solution is energetically and entropically favored verifying the fidelity of single‐phase solid solution formability toward unlocking novel energetic and physical properties for use in energy storage devices.^[^ ^4^, ^9^, ^41^ ^]^

Supporting the importance of entropic contributions toward single‐phase solid solution formability, the utilization of mechanochemistry was shown to controllably tune and modulate the degree of homogeneity with extension to diverse solid solution HEMs. For instance, select ratioed NiO:HEMN 1:1 and TiN:HEMO 1:1 (Figure S2B, Supporting Information), as well as LiCoO_2_:HEMO 1:5 and LiCoO_2_:HEMO:HEMN 2:5:5 (Figure S2C, Supporting Information) was synthesized via similar mechanochemical ball‐mill methods as previously mentioned. The XRD patterns of each HEM reveals the successful construction of a single‐phase solid solution with each select ratioed solid solution exhibiting five broad peaks that correspond to (111), (200), (220), (311), and (222) planes of a cubic F*m*3̅*m* rock‐salt phase. Additionally, peak shifts and broadening in the (200) reflection planes of each HEM compared to the respective precursor materials further indicates successful solid solution generation as represented in Figure S2 (Supporting Information). Further underlying the requirements of entropic effects toward single‐phase solid solution formability, NiO and TiN were mechanochemically co‐milled in a 1:1 ratio in similar fashion to that of HEMO:HEMN 1:1. The resulting NiO/TiN 1:1 material was analyzed using XRD (Figure S3, Supporting Information). Expectedly, the XRD pattern of NiO/TiN 1:1 reveals split peaks corresponding to the mixed phases of NiO and TiN respectively. The inability of NiO/TiN 1:1 to form a single‐phase highlight the lack of entropic influence, since, according to Equations (1)–(4), the possible number of random states and energy distributions must be tuned to produce a single‐phase solid solution. Therefore, maximizing the entropic effects by introducing and coordinating assorted anion and metallic cation sites is confirmed to be conducive toward generating a single‐phase solid solution.

As previously mentioned, a novel route toward preparing combined high entropy solid solutions under facile and ambient conditions is important toward preparing and leveraging diverse HEMs with unique physical and electrochemical capabilities. Mechanochemistry is commonly utilized as an effective synthesis method that provides instant high energy input enabling solid‐phase interactions toward single‐phase solid solution constructions under ambient conditions.^[^ ^42^, ^43^ ^]^ To display the requirement of the high energy environment provided by mechanochemistry toward producing a high entropy solid solution, a 1:1 ratio of HEMO and HEMN was physically mixed with a mortar and pestle. Naturally, the lack of high energy environments afforded by mechanochemical ball‐milling led to split peaks representing the individual lattice planes of the HEMO and HEMN respectfully (Figure S4, Supporting Information). Therefore, the feasibility of utilizing mechanochemistry toward generalized single‐phase solid solution formation as evidenced for HEMO:HEMN 1:1 (Figure 1A,B) is maintained.

The relative atomic metal composition for the 1:1 oxide/nitride solid solution was confirmed by Energy Dispersive X‐ray Spectroscopy (EDS) and represents the proportional distribution of each metal atom relative to the total number of metal atoms in the sample. This composition is within the accepted standard high entropy range^[^ ^44^, ^45^, ^46^ ^]^ and is outlined in Figure 1C. The full EDS spectra of HEMO:HEMN 1:1 is shown in Figure S5 (Supporting Information). Additionally, the relative atomic metal compositions deduced from Inductively Coupled Plasma Optical Emission Spectroscopy (ICP‐OES) for each as‐synthesized HEM are listed in (Table S1, Supporting Information). To better understand the morphology of the HEMO:HEMN 1:1 high resolution transmission electron microscopy (HRTEM) images were taken. HRTEM images reveal the 111‐reflection plane of a cubic F*m*3̅*m* rock‐salt phase (Figure 1D), which is in agreeance with XRD results (Figure 1B) and suggests successful integration of each respective HEM into a single‐phase solid solution. The chemical uniformity of the select ratioed high entropy oxide/nitride solid solutions is evidenced by High‐Angle Annular Dark Field (HAADF) used in Scanning Transmission Electron Microscopy (STEM) mode and EDS mapping, which confirms the homogeneous dispersion of each metal specie including V, Ti, Cr, Mo, Nb, Mn, Fe, Ni, Cu, Zn, O, and N for HEMO:HEMN 1:1 (Figure 1E). Only the diffraction rings from (111), (200), (220), and (311) of a cubic phase were identified in Fast Fourier Transform (FFT) pattern, validated the single‐phase of the HEMO:HEMN 1:1 solid solution. Additionally, the HAADF‐STEM image with selected area electron diffraction (SAED) (Figures S6A,B, Supporting Information) exhibits similar features to the FFT image in Figure 1D. Scanning Electron Microscopy (SEM) and EDS were used to confirm the homogeneous dispersion in HEMO:HEMN 2:1 (Figure S7, Supporting Information). Based on the single‐phase and peak shifts evidenced in the XRD results (Figure 1B), the homogeneity presented in the 1:1 oxide/nitride solid solution from EDS (Figure 1C,E), and the crystallinity presented in the HRTEM and HAADF‐STEM SAED images (Figure 1D; Figure S6A,B, Supporting Information), the combined features of HEMOs and HEMNs into a single high entropy solid solution is verified.

**Figure 2:**
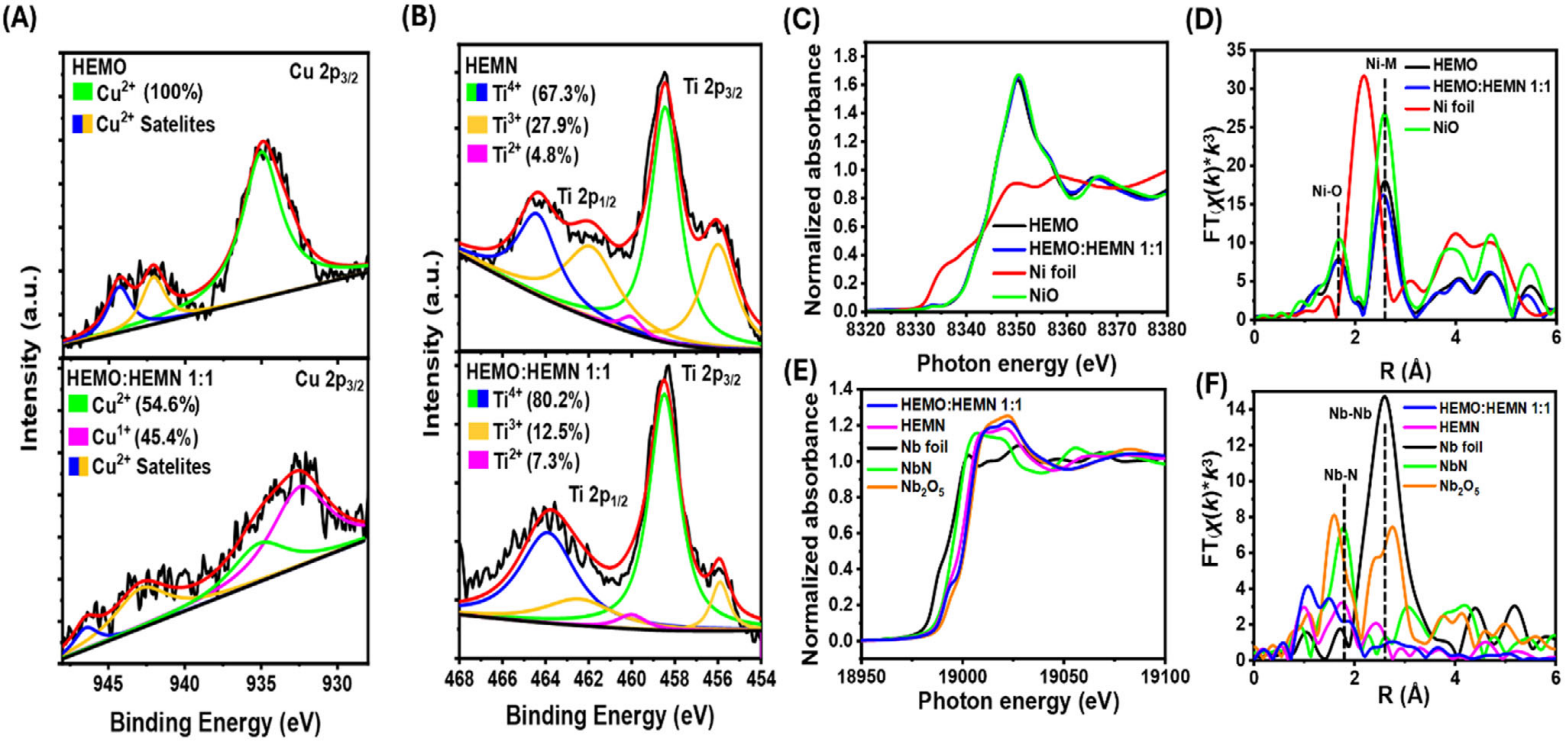
A) Cu 2p_3/2_ spectra of HEMO  and HEMO:HEMN 1:1. B) Ti 2p spectra of HEMO and HEMO:HEMN 1:1. C) XANES spectra at the Ni *K*‐edge and D) Fourier transformed EXAFS *k* ^3^‐weighted *χ*(*k*) data (∆*k* = 3–12 Å^−1^) in R‐space for Ni foil, NiO, HEMO, and HEMO:HEMN 1:1. E) XANES spectra at the Nb *K*‐edge and F) Fourier transformed EXAFS *k* ^3^‐weighted *χ*(*k*) data (∆*k* = 2.5–12 Å^−1^) in R‐space for Nb foil, NbN, Nb_2_O_3_, NbO_2_, Nb_2_O_5_, HEMN, and HEMO:HEMN 1:1. Data (C‐F) are not phase‐corrected.

The surface of the oxide/nitride high entropy solid solutions was analyzed with X‐ray Photoelectron Spectroscopy (XPS). The overall XPS survey spectrum of each HEM shows intense peaks at the specific binding energy regions that correspond to the transition metals for each respective HEM (Figure S8, Supporting Information). The generation of a successful oxide/nitride solid solution would be expected to reveal the metal cations coordinating with both O and N anion sites.^[^ ^47^ ^]^ This coordinative interaction would be indicated by a change in the oxidation state of the respective transition metals from each precursor HEM upon incorporation into the high entropy solid solution.^[^ ^48^ ^]^ Thus, the valence state of Cu (**Figure** **2**A) and Ti (Figure 2B) were analyzed. The Cu 2p_3/2_ spectrum of the HEMO features very weak satellite structures centered at 944.03 and 942.14 eV respectively and denote an oxidation state of 2^+^. Analysis of HEMO:HEMN 1:1 reveals a shift in the peak position of the Cu 2p_3/2_ satellite peaks from 942.14 eV in the HEMO to 941.93 eV. This decrease in the effective nuclear charge and a lower binding energy of the satellite peaks indicates the reduction of Cu from 2^+^ to 1^+^ during the formation of the oxide/nitride solid solution.^[^ ^49^, ^50^ ^]^ Apart from the satellite peaks, an overall change in the Cu^2+^ peak area (from 62.1% for HEMO to 24.3% for HEMO:HEMN 1:1) with an additional Cu^1+^ peak with an area of 45.4% is evidenced suggesting the reduction of Cu upon incorporation within the solid solution (Figure 2A). The reduction in Cu valence states upon HEMN incorporation into HEMO strongly supports the occurrence of nitrogen doping, as nitrogen acts as an electron donor when substituting for oxygen in the anion sublattice. Nitrogen anions possess lower electronegativity than oxygen, which increases the local electron density and stabilizes lower oxidation states of transition metals such as Cu.^[^ ^50^ ^]^ This observation is consistent with the expected electronic effects of nitrogen incorporation and supports its role in modifying the electronic structure of the solid solution. Additionally, these observations promote the validation of successful solid solution generation, since incorporating higher valence state ions into a solid solution, a charge compensation must occur to preserve the rock‐salt structure.^[^ ^51^ ^]^ For instance, if one component is reduced, another must be oxidized. Expectedly, the oxidation of Ti sites is observed when comparing the Ti 2p XPS results of the HEMN and solid solution, respectively (Figure 2B). For instance, the primary valence state of Ti in HEMN is 4^+^ and 3^+^ which corresponds to the coordination of Ti and N sites.^[^ ^52^ ^]^ This is upheld by the observation of peaks centered at 462.5 and 456.2 eV that correspond to the 3^+^ and 4^+^ valence states of Ti. Upon incorporation of the HEMN into the HEMO to form HEMO:HEMN 1:1, a decrease in peak area from 27.9% to 12.5% is observed for the 3^+^ valence state of Ti. Conversely, an increase in the peak area from 67.3% to 80.2% of the 4^+^ valence states at 464.1 and 459.2 eV respectively indicating the oxidation of Ti sites upon incorporation of the HEMN into HEMO to form the oxide/nitride solid solution (Figure 2B). The respective oxidation and reduction of the aforementioned metal species supports the notion of successful generation of a high entropy solid solution.

To provide more atomic‐level insights into the oxidation state and coordination environment of metal species, synchrotron X‐ray absorption near‐edge structure (XANES) and extended X‐ray absorption fine structure (EXAFS) measurements were performed at the National Synchrotron Light Source II. The Ni K‐edge XANES spectra (Figure 2C) show that the position of the rising edge and white line peaks for the HEMO and HEMO:HEMN 1:1 materials were similar to those of NiO standard,^[^ ^53^ ^]^ rather than Ni foil reference, confirming the predominant Ni^2+^ valence state. The Fourier transformed (FT) *k* ^3^‐weighted EXAFS for HEMO revealed Ni‐O correlations at 1.7 Å (non‐phase corrected), which is in agreement with the XAS results that have previously been reported for HEMO (MgNiCoCuZnO).^[^ ^54^, ^55^ ^]^ Interestingly, while the first‐sphere average distance is similar for HEMO and HEMO:HEMN samples, Ni─M (M = metal components in the high entropy materials) correlations exhibited noticeable differences (Figure 2D). The Ni─M correlations in HEMO:HEMN (2.52 Å) were found to be slightly shorter than that in the HEMO precursor (2.60 Å). This is somewhat expected based on the presence of additional multivalent metals in the structure, which likely produces strain leading to overall contraction and shorter Ni─M bonds.^[^ ^56^ ^]^ Additionally, this indicates a homogeneous atomic distribution in the solid solution due to shorter range, highly entropic atomic rearrangements of five metals with different atomic radii.^[^ ^57^ ^]^

Since our XPS results showed that some metals can undergo oxidation and reduction upon blending HEMN and HEMO systems, we performed additional XAS measurements for the Nb K‐edge. This element exhibits significant differences in its XANES features depending on its surrounding environment and oxidation state.^[^ ^58^ ^]^ In particular, the peak at ≈19 045 eV is typically observed only in the NbN standard, unlike in Nb₂O₅.^[^ ^59^ ^]^ Indeed, this peak appears in HEMN (Figure 2E), confirming the bonding of Nb^3^⁺ with nitrogen in this material. In contrast to HEMN, the mixed HEMO:HEMN 1:1 system lacks this feature, and its XANES spectrum closely resembles that of Nb₂O₅, corroborating the partial oxidation of Nb^3^⁺ to Nb⁵⁺ in the high‐entropy oxide/nitride solid solution. Specifically, the absence of the NbN‐specific feature at 19 045 eV in HEMO:HEMN 1:1 suggests that nitrogen coordination is no longer fully preserved in the same local geometry as in pure HEMN. However, the presence of a strong pre‐edge feature ≈18 981 eV, which is more intense than the Nb₂O₅ oxide reference, points to a partially retained nitrogen environment that induces asymmetry in the Nb coordination sphere. This increased asymmetry and distortion is consistent with nitrogen incorporation into the anion sublattice, where substitution of O^2^⁻ by N^3^⁻ modifies the local electronic environment and coordination geometry of Nb. Such substitution can lead to orbital rehybridization and localized states, supporting a mixed Nb–O/N coordination environment.^[^ ^60^ ^]^ These spectral features, when taken together, suggest that nitrogen is at least partially incorporated into the oxide lattice in the solid solution phase, contributing to the modified electronic structure. Specifically, this intense pre‐edge feature at ≈18 981 eV in the Nb K‐edge XANES spectrum of HEMO:HEMN 1:1 is attributed to the breaking of centrosymmetry, which leads to 5p‐4d orbital hybridization and allows dipolar 1s → 5p‐4d transitions.^[^ ^60^ ^]^ This indicates an increasing distortion of the local Nb environment with higher concentrations of metal‐oxygen sites in the HEMO:HEMN structure further supporting a mixed Nb–O/N coordination environment. Additionally, the differences observed in the XANES spectra of HEMN and HEMO:HEMN 1:1 reflect distinct Nb local environments, as further evidenced by the Fourier‐transformed EXAFS data (Figure 2F). Therefore, our XAS results support the successful incorporation of key elements into the high‐entropy oxide/nitride matrix and suggest the presence of a distinct local atomic and electronic structure in HEMO:HEMN.

### 2.2 Electrochemical Performance and Bandgap Characterization

The high redox‐site density of HEMO and the exceptional electronic conductivity of HEMN synergistically integrate within the HEMO:HEMN solid solution, endowing it with unique physicochemical properties that have the potential to enhance electrochemical energy storage performance. As an initial assessment, super capacitive behavior of the HEMO:HEMN 1:1 composite was tested in a conventional three‐electrode system with 1 m KOH as electrolyte (Figure S9, Supporting Information). The specific capacitance value for each HEM, was evaluated using Equation 5 where  C*_m_* (F g^−1^) represents the specific capacitance,  *I* represents the current, Δ*U* (V) is the potential window, *v* (mV s^−1^) is the scan rate, and *m* is the specific HEM mass casted on the working electrode.

(5) $$c_{m}=\frac{\int I\mathrm{dU}}{2mv\Delta U}$$

**Figure 3:**
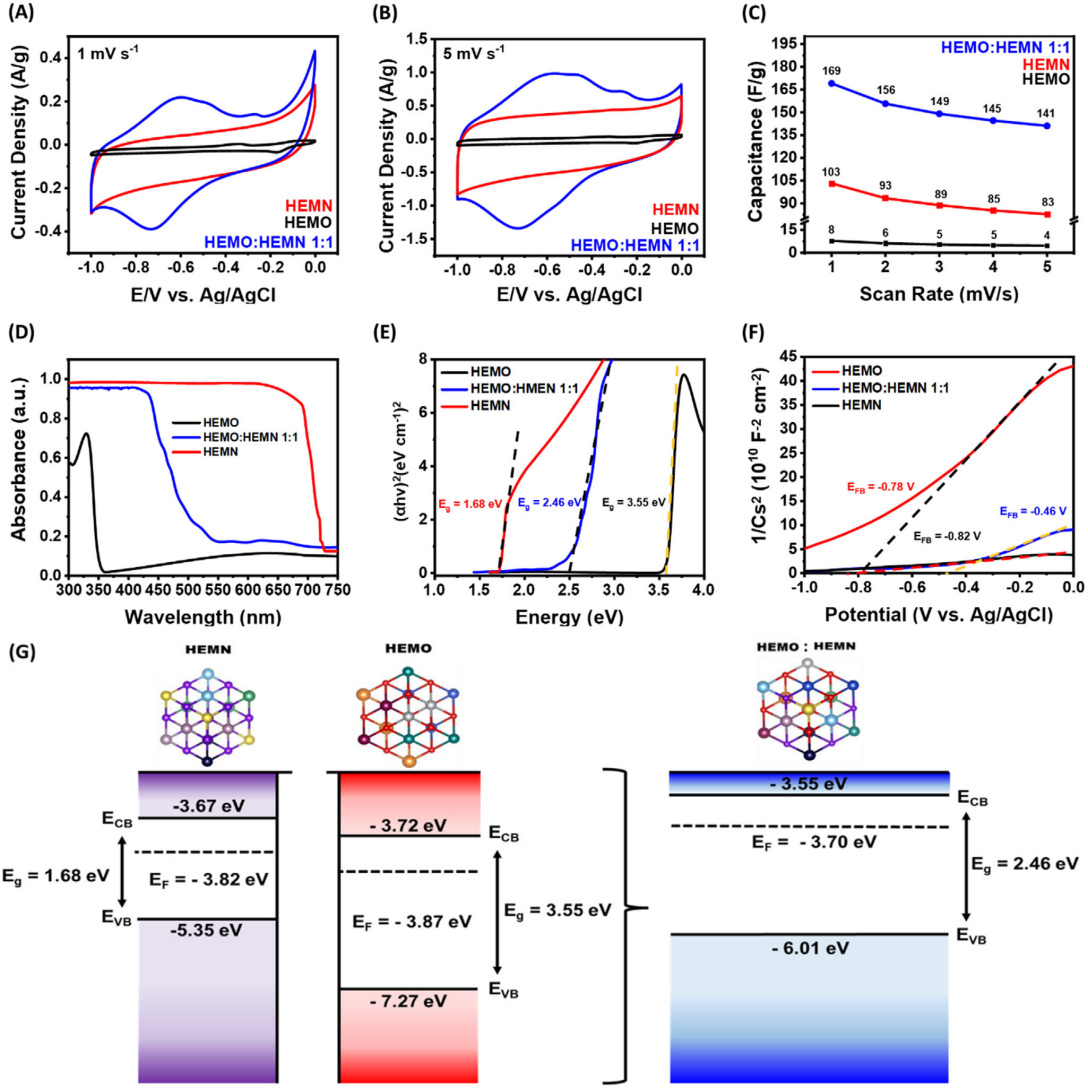
CVs of HEMO, HEMN, and 1:1 HEMO:HEMN in 1 m KOH at a scan rate of A) 1 mV s^−1^ and B) 5 mV s^−1^. C) Capacitance performance of HEMO, HEMN and HEMO:HEMN 1:1 at low scan rates (1–5 mV s^−1^). D) UV–vis spectra and E) Tuac Plots of HEMO, HEMN and HEMO:HEMN 1:1. F) Mott–Shottky Plots of HEMO, HEMN and HEMO:HEMN 1:1. G) Schematic illustration of charge transfer upon the contact formation of HEMO:HEMN 1:1.

As shown in **Figure** **3**A,B, HEMO exhibited negligible electrochemical activity despite its abundance of transition metal redox sites, primarily due to its low electronic conductivity. In contrast, HEMN, with its superior conductivity, demonstrated a significantly higher capacitance and a well‐defined rectangular CV curve, characteristic of electric double‐layer capacitor (EDLC) behavior. Notably, the CV curves of HEMO:HEMN 1:1 show a hybrid profile, characterized not only by quasi‐rectangular shapes, but also by prominent peaks that correspond to pseudocapacitance from the metal redox mechanism, highlighting the synergistic contributions of both HEMO and HEMN in the electrochemical performance. Specifically, the HEMO:HEMN 1:1 solid solution shows a significantly higher capacitance of 169 F g^−1^ at the scan rate of 1 mV s^−1^, surpassing both HEMO (8 F g^−1^) and HEMN (103 F g^−1^). When the scan rate increased to 5 mV s^−1^, the CV curve of HEMO:HEMN 1:1 retains its quasi‐rectangular shape and achieved a capacitance of 141 F g^−1^ (corresponding to 83.5% capacitance retention), which is much higher than the 83 and 5 F g^−1^ for HEMN and HEMO, respectively (Figure 3B,C).

The findings from these low scan rate assessments underscore the substantial enhancement in capacitance achieved in the oxide/nitride solid solutions compared to the precursor HEMs, a consequence possibly resulting from the concurrent utilization of EDL and redox capacitance. For comparison, the full CV curves of each HEM at scan rates of 1–10 mV s⁻¹ (−1.0 to 0 V vs Ag/AgCl) are shown in Figures S10–S13 (Supporting Information). Similar to HEMO:HEMN 1:1, HEMO:HEMN 2:1 (Figure S13, Supporting Information) displays quasi‐rectangular CVs with broad redox peaks: −0.38, −0.56, −0.38, −0.23, and −0.85 V, indicating combined EDL and pseudo‐capacitive behavior. Comparative analyses (Figures S14–S16, Supporting Information) reveal that both the 1:1 and 2:1 solid solutions outperform the HEMO and HEMN precursors in specific capacitance at scan rates of 1, 5, and 10 mV s⁻¹ with the similar performances of the 2:1 and 1:1 ratios being shown in Figure S17 (Supporting Information).

The possible inclusion of EDL and pseudocapacitive as a mechanism that leads to the enhancement of energy storage capabilities in the high entropy oxide/nitride solid solutions prompted further electrochemical analysis. To determine the charge storage mechanism, valence band (E_VB_), conduction band (E_CB_), and Fermi level (E_F_) positions of each HEM were assessed using UV–vis diffuse reflectance spectroscopy (UV–vis DRS) to determine the bandgap energy (E_g_) (Figure 3D) and Mott–Schottky (MS) analysis to evaluate the E_VB_, E_CB,_ and E_F_ positions based on the flat‐band potentials (E_FB_) (Figure 3F). E_g_ values were extracted by applying a Kubelka‐Munk function plot to the UV–vis spectra with direct transition for each HEM. The E_g_ values were identified to be 1.68, 2.46, and 3.55 eV for HEMN, HEMO:HEMN 1:1, and HEMO respectively (Figure 3E). The reduced bandgap energy of the HEMO:HEMN 1:1 solid solution is driven by a unique anion–cation co‐engineering strategy. In this approach, the incorporation of N‐doping from the HEMN component introduces new energy levels into the band structure, while the increased configurational entropy from cation doping induces variations in electronegativity, broadening the 3d states. This dual modification significantly narrows the bandgap compared to the HEMO, resulting in a substantial decrease in bandgap energy.^[^ ^18^ ^]^ Specifically, the incorporation of nitrogen into the HEMO:HEMN 1:1 solid solution introduces localized electronic states near the valence band, significantly increasing the density of states (DOS) close to the Fermi level. Nitrogen, as an electron donor, replaces oxygen in the anion sublattice, and its lower electronegativity compared to oxygen enhances the local electron density. This increase in electron density stabilizes lower oxidation states of transition metals, which contributes to the reduction of the material's bandgap. The nitrogen doping leads to hybridization between nitrogen 2p orbitals and the transition metal 3d orbitals, creating additional electronic states above the valence band maximum.^[^ ^18^ ^]^ These additional states facilitate electronic transitions, resulting in a narrowing of the bandgap. Thus, the narrow bandgap of HEMO:HEMN 1:1 compared to HEMO allows for easier electron excitation leading to higher electrical conductivity (HEMO:HEMN 1:1 = 4.2 × 10^−4^ S cm^−1^) which is more than 100 times higher than that of (HEMO = 2.6 × 10^−6^ S cm^−1^). Therefore, these results lead to the notion that the HEMO:HEMN solid solution shows improved charge transport which are important facets for high capacitance in energy storage devices.^[^ ^61^ ^]^

To further elucidate the electronic structure, the E_VB_, E_CB_, and E_F_ positions of the HEMs were determined through Mott–Schottky (MS) measurements at 1000 Hz by analysis of the flat‐band potentials (E_FB_) of each HEM, with the corresponding plots shown in Figure 3F. The linear range in the MS plots is analyzed further for each HEM with MS plots at 1000–500 Hz (Figure S18, Supporting Information). The E_FB_ values were identified as −0.94, −0.82, −0.78 V for HEMO:HEMN 1:1, HEMN, and HEMO respectively. The more negative E_FB_ in HEMO:HEMN 1:1 explains the enhanced capacitance, since a more negative flat‐band potential indicates greater band bending relative to the electrolyte, resulting in a stronger electric field. This stronger field provides a greater driving force for charge transfer, leading to higher capacitance compared to the starting HEMs.^[^ ^62^ ^]^ Additionally, the E_FB_ describes the voltage where the fermi level of the sample equilibrates with the redox couple (e.g., Ag/AgCl). Therefore, the E_FB_ can be used to calculate the fermi level (E_F_) of the sample in relation to the standard hydrogen electrode (SHE) and then converted to vacuum level (eV) for a total picture of the electronic states of each material.^[^ ^63^ ^]^ The E_F_ of each sample was calculated according to Equations (6) and (7).

(6) $$\begin{array}{ccc} E_{\mathrm{FB}}\left(V\;\mathrm{versus}\;\;\mathrm{SHE}\right) & = & E_{\mathrm{FB}}\;\left(V\;\mathrm{versus}\;\mathrm{Ag}/\mathrm{AgCl}\right)+0.205\;\left(V\right)\\ & & =E_{F}\left(V\;\mathrm{versus}\;\;\mathrm{SHE}\right) \end{array}$$

(7) $$E_{F}\;\left(\mathrm{eV}\right)=E_{F}\;\left(V\;\mathrm{versus}\;\mathrm{SHE}\right)+4.44\;V$$

The E_F_ of each HEM were calculated to be −3.82, −3.87, and −3.70 eV for HEMN, HEMO, and HEMO:HEMN 1:1 respectively. As noted by the positive slope in the MS plots (Figure 3F) and the E_g_ values, each HEM can be described as an n‐type semiconductor. For n‐type semiconductors, the E_F_ lies ≈0.1 to 0.2 eV below the E_CB_.^[^ ^64^ ^]^ Based on this approximation, the E_CB_ potentials are determined to be −3.67, −3.72, and −3.55 eV for HEMN, HEMO, and HEMO:HEMN 1:1 respectively. Using the relationship between E_g_ and E_CB_, the E_VB_ was determined to be −5.35, −7.27, and −6.01 eV for HEMN, HEMO, and HEMO:HEMN 1:1 respectively. The resulting band structures of the HEMs are presented in Figure 3G. Notably, after inclusion of HEMN into HEMO, the E_VB_ potential of the HEMO:HEMN 1:1 system is positively shifted by 1.26 eV relative to that of the HEMO system, while the E_CB_ edge potential is shifted by 0.17 eV. Additionally, the E_F_ of HEMO:HEMN 1:1 shifted by 0.17 eV indicating a more electron rich environment favoring pseudocapactive mechanisms for charge storage.^[^ ^65^ ^]^ Thus, the shifts of both the E_VB_ and E_CB_ toward more positive potentials indicate that the HEMO:HEMN 1:1 system possesses enhanced redox capabilities. This conclusion is further supported by the amplified redox peak intensities observed in the cyclic voltammetry analysis (Figure 3A,B).

**Figure 4:**
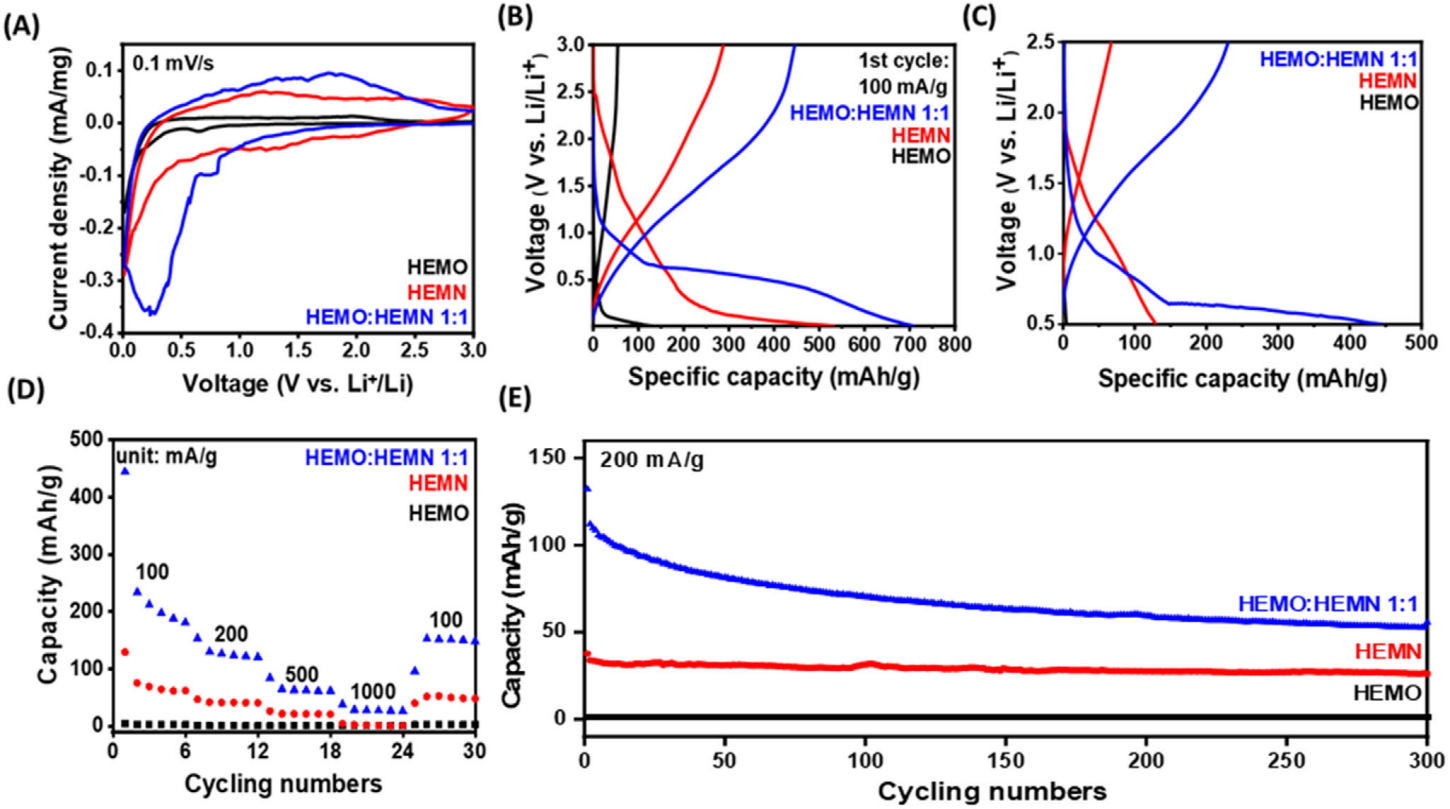
Electrochemical characterizations for HEMO, HEMN, and HEMO:HEMN 1:1: A) CV curves at 0.1 mV s^−1^ from 0.01 to 3 V. B) Galvanostatic charge/discharge curves from 0.01 to 3 V. C) Galvanostatic charge/discharge curves from 0.5 to 2.5 V. D) Rate performance from 0.5 to 2.5 V. E) Long‐term cycling performance at 200 mA g^−1^ from 0.5 to 2.5 V.

To further demonstrate the advantage of the HEMO:HEMN solid solution in electrochemical energy storage application, the electrochemical capabilities of each HEM were also analyzed utilizing lithium‐ion battery (LIB) applications. Similar to their application in supercapacitors, TMNs offer stable chemical properties and high electronic conductivity but lack sufficient active lithium storage sites, while TMOs provide abundant lithium‐ion storage through reversible redox reactions, though they suffer from low electrical conductivity and structural stability during cycling.^[^ ^15^ ^]^ To harness their potential for electrochemical energy storage and circumvent the insufficiencies of low conductivity and lithium‐ion storage sites, intricate nanoengineering that utilizes a synergistic and combinatory arrangement of TMOs and TMNs is imperative. In the battery CV tests, both HEMO and HEMN exhibit a reductive peak below 0.1 V, corresponding to electrolyte decomposition. Notably, the HEMO:HEMN 1:1 displays a distinct peak at ≈0.3 V, indicating enhanced utilization of HEMO's conversion reaction with lithium ions, as shown in **Figure** **4**A. Additionally, the area encompassed within the CV plots is greatest in HEMO:HEMN 1:1 indicating more lithium‐ion storage capabilities compared to the starting HEMs. The charge–discharge curves of HEMN displayed a sloping profile above 0.5 V versus Li/Li⁺, characteristic of pseudocapacitive charge storage involving distributed redox sites (Figure 4B). In contrast, both HEMO and HEMN exhibited pronounced discharge plateaus below 0.5 V, indicative of conversion‐type reactions. The charge–discharge profile of the HEMO:HEMN composite featured both sloped regions and distinct plateaus, reflecting a combination of pseudocapacitive and conversion‐type energy storage mechanisms. The enhanced lithium‐ion storage can be attributed the smaller bandgap and a higher E_F_​ of HEMO:HEMN 1:1, as a higher E_F_ and a lower bandgap may facilitate efficient electron transfer, which is critical for lithium‐ion storage in this system.^[^ ^66^ ^]^ Moreover, the high‐entropy solid solution (HEMO:HEMN 1:1) demonstrated superior performance with a discharge capacity of 709 mAh g^−1^ at 100 mA g^−1^, surpassing HEMO (130 mAh g^−1^) and HEMN (532 mAh g^−1^) at the same testing conditions (Figure 4B).To avoid excessive structural collapse resulting from overly thorough conversion reactions, the charge‐discharge voltage from 0.5 to 2.5 V Li^+^/Li was also collected (Figure 4C–E). The rate capability tests within this voltage window reveal that HEMO:HEMN 1:1 exhibits discharge capacities of 444, 153, 84, and 27 mAh g^−1^ at 100, 200, 500, and 1000 mA g^−1^, respectively, surpassing those of HEMO and HEMN (Figure 4D). Additionally, as shown in Figure 4E, HEMO:HEMN 1:1 also demonstrates favorable cycling stability compared to HEMN and HEMO over 300 cycles. At the 300^th^ cycle, the specific capacity of HEMO:HEMN 1:1 is 53 mAh g^−1^, which is still much higher than the HEMN (23 mAh g^−1^) and HEMO (1 mAh g^−1^) starting materials. Thus, these results and the methods presented herein pave the way for future research into the regulation and modulation of diverse HEMs toward enhanced energy storage and electrochemical capabilities. Additionally, the findings from these electrochemical evaluations underscore the substantial enhancement in supercapacitors and lithium‐ion batteries achieved in the oxide/nitride solid solutions compared to the employed starting HEMs.

## 3 Conclusion

In summary, high‐entropy solid solutions with controlled composition ratios were successfully achieved through mechanochemical synthesis at ambient temperature. By introducing HEMN into HEMO lattices, a significant reduction in the bandgap from 3.55 eV (HEMO) to ≈2.46 eV was achieved, which is driven by a unique cation‐anion co‐engineering strategy. This strategy combines cation doping‐induced configurational entropy and anion doping from HEMN to further reduce the bandgap, significantly enhancing the material's electronic conductivity. Leveraging the synergistic interplay between electrochemical double‐layer (EDL) and pseudo‐capacitance redox reactions, the 1:1 ratio high‐entropy oxide/nitride solid solution (HEMO:HEMN 1:1) exhibits a tenfold increase in capacitance and capacity compared to HEMO in supercapacitor and lithium‐ion battery tests, respectively. The outstanding supercapacitor performance of the oxide/nitride solid solution can be attributed to the cooperative synergy among the constituent HEMs and their uniform atomic distribution. Furthermore, this methodology shows promise in paving the way to create a wide range of diverse high entropy solid solution configurations that combine cation and anion doping for tailored bandgap and electronic properties. The observed increase in capacitance, enhanced performance in battery tests, and the feasibility of generating solid solution HEMs position these materials as promising candidates for various practical electrochemical applications. While the battery performance could be further improved by combining the bandgap engineering with other strategies such as morphology and pore structure control, further investigation into their potential uses in energy storage and other domains is encouraged.

## 4 Experimental Section

### Materials

All the chemical reagents used in this work were of analytical grade. Cobalt chloride, anhydrous (99.0% CoCl_2_, Acros, USA), chromium chloride, anhydrous (99.0% CrCl_3_, Acros, USA), copper chloride, anhydrous (99.0% CuCl_2_, Acros, USA), magnesium chloride, anhydrous (99.0% MgCl_2_, Alfa Aesar, USA), molybdenum chloride, anhydrous (99.0%, MoCl_5_, Acros, USA), niobium chloride, anhyrous (99.8%, NbCl_5_, Sigma–Aldrich, USA), nickel chloride, anyhrous (99.0%, NiCl_2_, Alfa Aesar, USA), titanium chloride (98% TiCl4, Acros, USA), Urea (99.5%, Acros, USA), vanadium chloride, anhydrous (99.9%, VCl_3_, Sigma–Aldrich, USA), and zinc chloride, anhydrous (98% ZnCl_2_, Acros, USA), were all used as received without further purification.

### HEM Synthesis

Synthesis of the HEMN was done using similar methods previously outlined by group.1 In a typical synthesis, equimolar (0.5 mmol) VCl3 (Sigma–Aldrich, 99.9%), CrCl3 (Acros, 99%), NbCl5 (Sigma–Aldrich, 99.8%), MoCl5 (Acros, 99%), and TiCl4 (Acros, 98%) were mixed with 25 mmol urea (Acros, 99.5%). The metal chlorides and urea were added to a commercially available zirconia vial reactor along with three zirconia balls. The reactor was placed in a high‐speed vibrating ball miller (1200 rounds min^−1^, 300 W motor power) and the mixture was ball milled for 30 min. The resulting gel‐like product was pyrolyzed at 800 °C (heating rate: 5 K min^−1^) for 3 h under an inert N2 atmosphere to afford the HEMN.

Synthesis of the HEMO was done using equimolar (0.5 mmol) CoCl2 (Acros, 99%), CuCl2 (Acros, 99%), MgCl2 (Alfa Aesar, 99%) NiCl2 (Alfa Aesar, 99%), and ZnCl2 (Acros, 98%). The metal chlorides were added to a commercially available zirconia vial reactor along with three zirconia balls. The reactor was placed in a high‐speed vibrating ball miller (1200 rounds min^−1^, 300 W motor power) and the mixture was ball milled for 30 min. The resulting gel‐like product was pyrolyzed at 900 °C (heating rate: 5 K min^−1^) for 3 h under an inert ambient atmosphere to afford the HEMO.

Synthesis of the HEMO:HEMN materials was done using select molar ratio amounts (1:1 vs 2:1) of the HEMO and HEMN added to a stainless‐steel ball‐milling reactor jar with three stainless steel planetary balls. The reactor was placed in a high‐speed vibrating ball miller (1200 rounds min^−1^, 300 W motor power) and the mixture was ball milled for 30 min to afford the select ratioed oxide/nitride solid solution. Similar procedure was used to synthesize select molar.

### Structural Characterization

X‐ray diffraction (XRD) data were recorded using the PANalytical Empyrean diffractometer at 45 kV and 40 mA. Diffraction patterns were recorded in the range of 5–80° with a 0.02 step size and λ = 0.1 540 598 nm. X‐ray photoelectron spectroscopy (XPS) was performed with a Thermo Scientific Model K‐Alpha XPS Instrument, which uses micro‐focused monochromatic Al Kα X‐rays (1486.6 eV) that are focused 30 to 400 microns. For the XPS experiment, the analysis of samples was conducted with a 400 µm X‐ray spot size to allow for maximum signal to be obtained. Additionally, the instrument uses a hemispherical electron energy analyzer equipped with a 128‐channel electron detection system. The base pressure in the analysis chamber was typically 2 × 10^−9^ mbar or lower. The resulting XPS spectra were analyzed using the CasaXPS and XPSPEAK41 programs. Further chemical composition analysis on the atomic percentage of the transition metals within each of the samples was determined by inductively coupled plasma optical emission spectrometry (ICP‐OES, Optima 7000DV, PerkinElmer, USA).

The Transmission electron microscopy (TEM) sample was prepared by dispersing 5 mg of sample in ethanol, assisted by sonication for 30 min. After that, the sample was dropped on the copper grid and dried in vacuum. Then, TEM was performed using a Zeiss Libra200 electron microscope operated at 200 kV. The scanning electron microscopy images (SEM) and energy‐dispersive X‐ray spectroscopy (EDS) mapping images were recorded on a Hitachi S‐4800 microscope operated at an accelerating voltage of 15.0 kV. High‐angle annular dark‐field scanning tunneling electron microscopy (HAADF‐STEM) images were collected on a Fisher Scientific Spectra 300 electron microscope operating at 200 kV.

X‐ray absorption spectroscopy (XAS) measurements were performed at the 6‐BM beamline (NSLS‐II) for the Ni and Nb K‐edge of the produced high‐entropy materials. All samples were energy calibrated to a respective metal foil reference. All samples were prepared as pellets by mixing with varying amounts of boron nitride and contained in polyimide foil. Measurements were performed in transmission mode. Data normalization and the Fourier transformed EXAFS analysis was carried out using the Athena software package.^[^ ^67^ ^]^ The smooth background was subtracted using the AUTOBK code with *R*_bkg_ equals to 1.0 to obtain the normalized *χ*(k) data.

### Electrochemical Performance and Bandgap Characterization

The materials were prepared for electrochemical analysis by weighing 7 mg into a vial after grating the sample with a 300 mesh sieve. Next, a 0.5 mL solution consisting of 4 mg mL^−1^ carbon black and 2 mg mL^−1^ nafion binder in isopropyl alcohol was added to the vial with 7 mg of sample and then sonicated for 30 min. Then, 10 µL of the resulting solution was drop casted onto a glassy carbon electrode. The supercapacitive behavior of each material was then evaluated using a conventional three‐electrode cell with 1m KOH as an electrolyte, a Pt wire counter electrode, and an Ag/AgCl reference electrode.

UV–vis diffuse reflectance spectra (UV–vis DRS) were obtained on a Fisher Scientific Evolution 300 UV–vis spectrometer equipped with a Harrick Scientific praying manting diffuse reflectance cell. Spectra were collected from 300 to 750 nm, with 2 nm steps. The reference spectrum for total reflectance was measured against a MgO standard. To generate samples, the powders were pelletized into a 1 cm diameter disk. Bandgaps were calculated by generating Tuac plots from the UV–vis spectra and fitting the linear region in the onset of absorption and extrapolating to the *x*‐intercept. Specific conductivity measurements were carried out using the two‐electrode method under a pressure of 3T.

Mott–Schottky analysis was done under the same conditions as the CV tests with 1 m KOH as an electrolyte, a Pt wire counter electrode, and an Ag/AgCl reference electrode. The materials were prepared for Mott–Schottky analysis by weighing 7 mg into a vial after grating the sample with a 300 mesh sieve. Next, a 0.5 mL solution consisting of 2 mg mL^−1^ nafion binder in isopropyl alcohol was added to the vial with 7 mg of sample and then sonicated for 30 min. Then, 10 µL of the resulting solution was drop casted onto a glassy carbon electrode. The Mott–Schottky measurements were done under the impedance‐potential mode from 0 to −1 V with an amplitude of 0.005 and a frequency of 1000 and 500 Hz.

The electrochemical lithium‐ion battery performance of the as‐prepared HEMO, HEMN and HEMO:HEMN 1:1 solid solution were tested by half cells with lithium foil as the counter electrode. The slurry was prepared by mixing 90 wt.% of active material, 5 wt.% of carbon black as conducting agent, and 5 wt.% of polyvinylidene fluoride in N‐methylpyrrolidinone as binder. The mixture was then casted uniformly on aluminum foil and dried under vacuum at 100 °C overnight. After drying, the electrode was punched into circular discs with a mass loading of ≈5 mg cm^−2^. Next, CR2032‐type half batteries were assembled in an argon‐filled glovebox with Celgard 2320 film as separator, and 1.2 m LiPF6 in a mixture of ethylene carbonate and dimethyl carbonate (3:7 by volume) used as the electrolyte. The long cycle performance and rate performance evaluations were conducted on a Neware battery test system at different current densities within a potential range of 0.01–3.0 or 0.05–2.5 V. The Cyclic voltammetry test was carried out on BioLogic system to examine the electrode reaction under the scan rate of 0.1 mV s^−1^.

## Conflict of Interest

The authors declare no conflict of interest.

## Supporting information



Supporting Information

## Data Availability

The data that support the findings of this study are available from the corresponding author upon reasonable request.
