# Supplementary material for: A General Strategy for Bandgap Engineering Via Anion‐Lattice Doping in High‐Entropy Oxides
Source: Adv Sci (Weinh). 2025 Jun 19;12(34):e05789. doi: 10.1002/advs.202505789 (PMC12442597; doi:10.1002/advs.202505789)
Supplement: Supplementary file 1 — Supporting Information [file ADVS-12-e05789-s001.pdf]

ADVANCED SCIENCE
Open Access

Supporting Information

for *Adv. Sci.*, DOI 10.1002/advs.202505789

A General Strategy for Bandgap Engineering Via Anion-Lattice Doping in High-Entropy Oxides

Kevin Siniard, Juntian Fan, Meijia Li, Qingju Wang, Alexander S. Ivanov, Tao Wang and Sheng Dai**

Supporting Information

A General Strategy for Band-Gap Engineering via Anion-Lattice Doping in High-Entropy Oxides

Kevin Siniard, Juntian Fan, Meijia Li, Qingju Wang, Alexander S. Ivanov, Tao Wang, and Sheng Dai**

K. Siniard, Dr. Q. Wang, Dr. S. Dai
Department of Chemistry, Institute for Advanced Materials and Manufacturing
University of Tennessee, Knoxville
Knoxville, TN, 37996, USA.

Dr. J. Fan, Dr. M. Li, Dr. A. S. Ivanov, Dr. T. Wang, Dr. S. Dai
Chemical Sciences Division
Oak Ridge National Laboratory
Oak Ridge, TN, 37831, USA.
*E-mail: wangt@ornl.gov; dais@ornl.gov

Materials and Chemicals

All the chemical reagents used in this work were of analytical grade. Cobalt chloride, anhydrous (99.0 % $CoCl_2$, Acros, USA), chromium chloride, anhydrous (99.0% $CrCl_3$, Acros, USA), copper chloride, anhydrous (99.0% $CuCl_2$, Acros, USA), magnesium chloride, anhydrous (99.0% $MgCl_2$, Alfa Aesar, USA), molybdenum chloride, anhydrous (99.0%, $MoCl_5$, Acros, USA), niobium chloride, anhyrous (99.8%, $NbCl_5$, Sigma-Aldrich, USA), nickel chloride, anyhrous (99.0%, $NiCl_2$, Alfa Aesar, USA), titanium chloride (98% TiCl4, Acros, USA), Urea (99.5%, Acros, USA), vanadium chloride, anhydrous (99.9%, VCl_3, Sigma-Aldrich,USA), and zinc chloride, anhydrous (98% $ZnCl_2$, Acros, USA), were all used as received without further purification.

Characterization and Experimental Setup

- **X-ray diffraction (PXRD)**

X-ray diffraction (XRD) data were recorded using the PANalytical Empyrean diffractometer at 45 kV and 40 mA. Diffraction patterns were recorded in the range of 5-80° with a 0.02 step size and λ = 0.1540598 nm. Rietveld refinement of the XRD data was performed using the GSAS-II software package. The refinement was initiated using a standard crystallographic information file (CIF) of CoO with the Fm-3m space group as the starting structural model. The background, scale factor, lattice parameters, etc., were refined sequentially to achieve the best fit between the experimental and calculated patterns.

- **X-ray photoelectron spectroscopy (XPS)**

X-ray photoelectron spectroscopy (XPS) was performed with a Thermo Scientific Model K-Alpha XPS Instrument, which uses micro-focused monochromatic Al Kα X-rays (1486.6 eV) that are focused 30 to 400 microns. For the XPS experiment, the analysis of samples was conducted with a 400 μm X-ray spot size to allow for maximum signal to be obtained. Additionally, the instrument uses a hemispherical electron energy analyzer equipped with a 128-channel electron detection system. The base pressure in the analysis chamber was typically 2×10^{-9} mbar or lower. The resulting XPS spectra were analyzed using the CasaXPS and XPSPEAK41 programs.

- **Inductively coupled plasma optical emission spectrometry (ICP-OES)**

Further chemical composition analysis on the atomic percentage of the transition metals within each of the samples was determined by inductively coupled plasma optical emission spectrometry (ICP-OES, Optima 7000DV, PerkinElmer, USA).

- **Transmission electron microscopy (TEM)**

Transmission electron microscopy (TEM) sample was prepared by dispersing 5 mg of sample in ethanol assisted by sonication for 30 minutes. After that, the sample was dropped on the

copper grid and dried in vacuum. Then, TEM was performed using a Zeiss Libra200 electron microscope operated at 200 kV. The scanning electron microscopy images (SEM) and energy-dispersive X-ray spectroscopy (EDS) mapping images were recorded on Hitachi S-4800 microscope operated at an accelerating voltage of 15.0 kV. High-angle annular dark-field scanning tunneling electron microscopy (HAADF-STEM) images were collected on a Fisher Scientific Spectra 300 electron microscope operating at 200 kV.

- **Energy-dispersive X-ray spectroscopy (EDS)**

The energy-dispersive X-ray spectroscopy (EDS) mapping images were recorded on Hitachi H-7700 microscope with an acceleration voltage of 100 kV.

- **Cyclic voltammetry (CV) test for supercapacitance**

The materials were prepared for electrochemical analysis by weighing 7mg into a vial after grating the sample with a 300 mesh sieve. Next, a 0.5 mL solution consisting of 4 mg / mL carbon black and 2 mg / mL nafion binder in isopropyl alcohol was added to the vial with 7mg of sample and then sonicated for 30 minutes. Then, 10 μL of the resulting solution was drop casted onto a glassy carbon electrode. The supercapacitive behavior of each material was then evaluated using a conventional three-electrode cell with 1M KOH as an electrolyte, a Pt wire counter electrode, and a Ag/AgCl reference electrode.

- **UV-Vis diffuse reflectance spectroscopy (UV-Vis DRS)**

UV-Vis diffuse reflectance spectra (UV-Vis DRS) were obtained on a Fisher Scientific Evolution 300 UV–visible spectrometer equipped with a Harrick Scientific praying manting diffuse reflectance cell. Spectra were collected from 300 to 750 nm, with 2 nm steps. The reference spectrum for total reflectance was measured against a MgO standard. To generate samples, the powders were pelletized into a 1 cm diameter disk. Band gaps were calculated by generating Tuac plots from the UV-Vis spectra and fitting the linear region in the onset of absorption and extrapolating to the *x*-intercept.

- **Mott Schottky (MS) analysis**

Mott Schottky analysis was done under the same conditions as the CV tests with 1M KOH as an electrolyte, a Pt wire counter electrode, and an Ag/AgCl reference electrode. The materials were prepared for Mott Schottky analysis by weighing 7mg into a vial after grating the sample with a 300 mesh sieve. Next, a 0.5 mL solution consisting of 2 mg / mL nafion binder in isopropyl alcohol was added to the vial with 7mg of sample and then sonicated for 30 minutes. Then, 10 μL of the resulting solution was drop casted onto a glassy carbon electrode. The Mott Schottky measurements were done under the impedance-potential mode from 0 to -1 V with an amplitude of 0.005 and a frequency of 1000Hz and 500 Hz.

- **Lithium-ion battery (LIB) performance test**

The electrochemical lithium-ion battery performance of the as-prepared HEMO, HEMN and HEMO:HEMN 1:1 solid solution were tested by half cells with lithium foil as the counter electrode. The slurry was prepared by mixing 90 wt % of active material, 5 wt % of carbon black as conducting agent, and 5 wt % of polyvinylidene fluoride in N-methylpyrrolidinone as binder. The mixture was then casted uniformly on aluminum foil and dried under vacuum at 100 °C overnight. After drying, the electrode was punched into circular discs with a mass loading of ~5 mg cm^{-2} . Next, CR2032-type half batteries were assembled in an argon-filled glovebox with Celgard 2320 film as separator, and 1.2 M LiPF6 in a mixture of ethylene carbonate and dimethyl carbonate (3:7 by volume) used as the electrolyte. The long cycle performance and rate performance evaluations were conducted on a Neware battery test system at different current densities within a potential range of 0.01–3.0 V or 0.05–2.5 V. The Cyclic voltammetry test was carried out on BioLogic system to examine the electrode reaction under the scan rate of 0.1 mV s^{-1}.

- **X-ray absorption spectroscopy (XAS)**

X-ray absorption spectroscopy (XAS) measurements were performed at the 6-BM beamline (NSLS-II) for the Ni and Nb K-edge of the produced high entropy materials. All samples were energy calibrated to a respective metal foil reference. All samples were prepared as pellets by mixing with varying amount of boron nitride and contained in polyimide foil. Measurements were performed in transmission mode. Data normalization and the Fourier transformed EXAFS

analysis was carried out using the Athena software package (Ravel, B. & Newville, M. ATHENA, ARTEMIS, HEPHAESTUS: data analysis for X-ray absorption spectroscopy using IFEFFIT. *J. Synchrotron Radiat.* **12**, 537−541 (2005)). The smooth background was subtracted using the AUTOBK code with R_{bkg} equals to 1.0 to obtained the normalized χ(k) data. The k^3-weighted $\chi(k)$ data were obtained by the Fourier transform by applying a Hanning window (dk=1*dk*=1 $Å^{-1}$). Only data preprocessing and visualization were performed; no EXAFS fitting was conducted in this study.

Synthesis of High Entropy Precursors

- **Synthesis of HEMN ($VTiCrMoNbN_x$)**

Synthesis of the HEMN was done using similar methods previously outlined by our group.[1] In a typical synthesis, equimolar (0.5 mmol) VCl_3 (Sigma-Aldrich, 99.9%), $CrCl_3$ (Acros, 99%), $NbCl_5$ (Sigma-Aldrich, 99.8%), $MoCl_5$ (Acros, 99%), and $TiCl_4$ (Acros, 98%) were mixed with 25 mmol urea (Acros, 99.5%). The metal chlorides and urea were added to a commercially available zirconia vial reactor along with three zirconia balls. The reactor was placed in a high-speed vibrating ball miller (1200 rounds min^{-1}, 300 W motor power) and the mixture was ball milled for 30 min. The resulting gel-like product was pyrolyzed at 800 °C (heating rate: 5 K min^{-1}) for 3 h under an inert N_2 atmosphere to afford the HEMN.

- **Synthesis of HEMO ($MgCoNiCuZnO_x$)**

Synthesis of the HEMO was done using equimolar (0.5 mmol) $CoCl_2$ (Acros, 99%), $CuCl_2$ (Acros, 99%), $MgCl_2$ (Alfa Aesar, 99%) $NiCl_2$ (Alfa Aesar, 99%), and $ZnCl_2$ (Acros, 98%). The metal chlorides were added to a commercially available zirconia vial reactor along with three zirconia balls. The reactor was placed in a high-speed vibrating ball miller (1200 rounds min^{-1}, 300 W motor power) and the mixture was ball milled for 30 min. The resulting gel-like product was pyrolyzed at 900 °C (heating rate: 5 K min^{-1}) for 3 h under an inert ambient atmosphere to afford the HEMO.

Mechanochemistry-driven oxide/nitride solid solution construction

- **Synthesis of HEMO:HEMN 1:1, HEMO:HEMN 2:1, TiN:HEMO 1:1, NiO:HEMN 1:1, $LiCoO_2$:HEMO 1:5, $LiCoO_2$:HEMO:HEMN 2:5:5, NiO:TiN 1:1**

Synthesis of the HEMO:HEMN materials was done using select molar ratio amounts (1:1 vs 2:1) of the HEMO and HEMN added to a stainless-steel ball-milling reactor jar with three stainless steel planetary balls. The reactor was placed in a high-speed vibrating ball miller (1200 rounds min^{-1}, 300 W motor power) and the mixture was ball milled for 30 min to afford the select ratioed oxide/nitride solid solution. Similar procedure was used to synthesize select molar ratio amounts (1:1, 1:5, and 2:5:5) of NiO:TiN 1:1, TiN:HEMN 1:1, TiN:HEMO 1:1, LiCoO2:HEMO 1:5, and LiCoO2:HEMO:HEMN 2:5:5.

Supplementary tables and figures

Table S1. Relative Atomic Metal Compositions Determined by ICP-OES for Each HEM, Representing the Ratio of Each Metal Element Relative to the Total Metal Content in the Sample.

Sample	Cr	Cu	Ti	V	Mo	Co	Nb	Zn	Mg	Ni
**HEMO**	-	19.1	-	-	-	22.5	-	19.6	18.0	20.8
**HEMN**	18.4	-	19.6	18.7	20.9	-	22.4	-	-	-
**1:1**	10.5	9.2	10.2	11.6	9.6	8.9	9.5	9.3	11.6	9.6
**2:1**	5.8	13.2	6.3	6.0	6.5	15.3	6.7	13.7	12.4	14.1

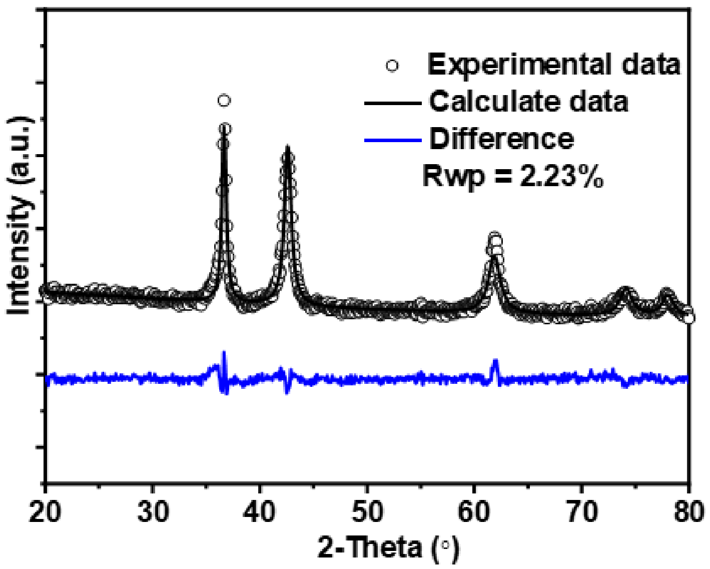

Figure S1. Rietveld refinement for the XRD peaks of HEMN:HEMO 1:1.

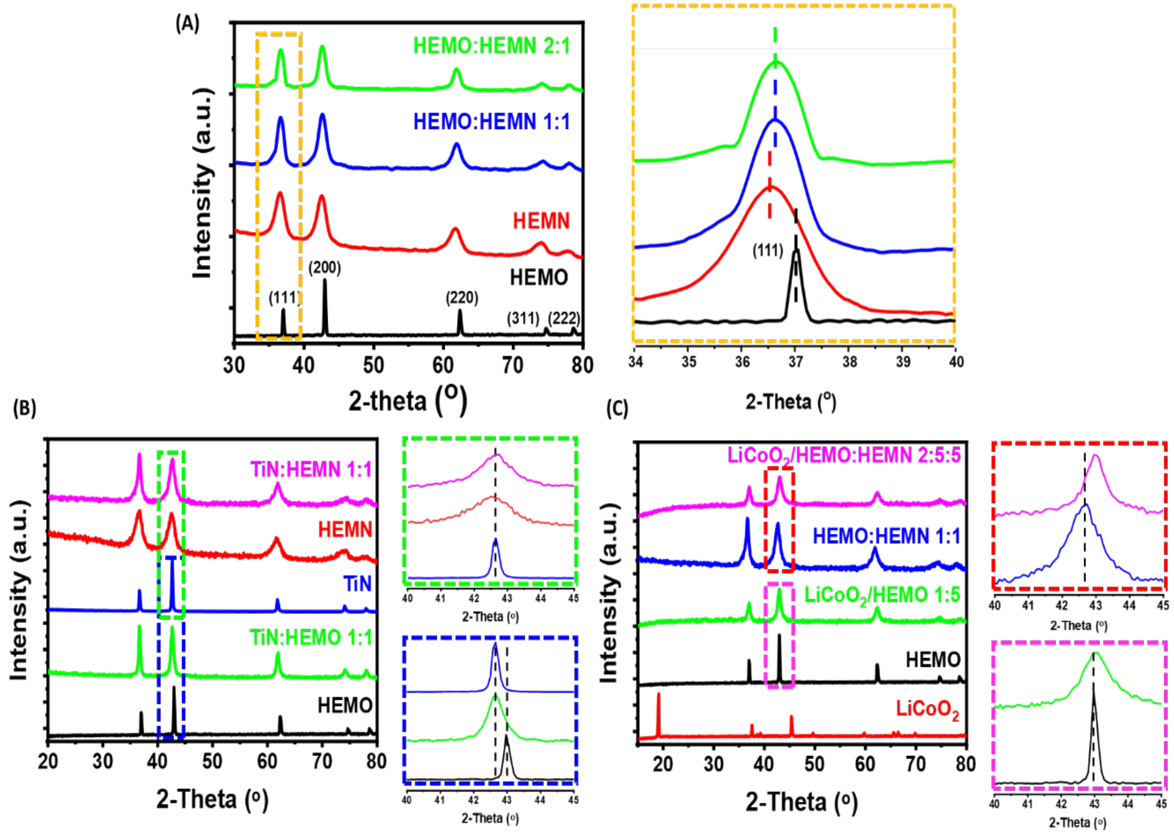

Figure S2. (A) XRD analysis of HEMO, HEMN, HEMO:HEMN 1:1, and 2:1 solid solutions with enlarged area marked in orange (B) XRD analysis of NiO:HEMN 1:1 and TiN:HEMO 1:1with enlarged areas marked in green and blue. XRD analysis of LiCoO2:HEMO 1:5 and LiCoO2:HEMO:HEMN 2:5:5 with enlarged areas marked in red and pink.

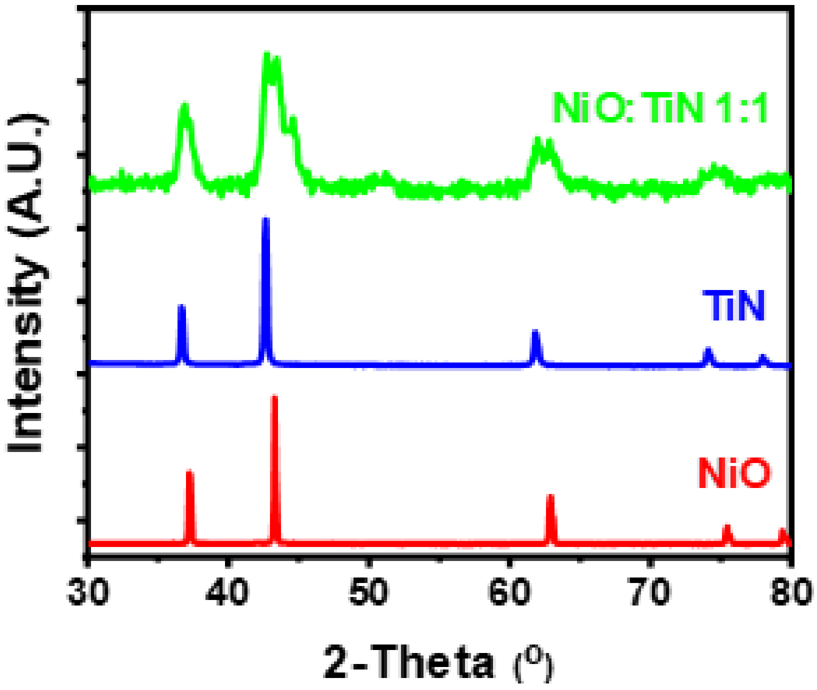

Figure S3. XRD patterns of NiO, TiN, and NiO:TiN 1:1.

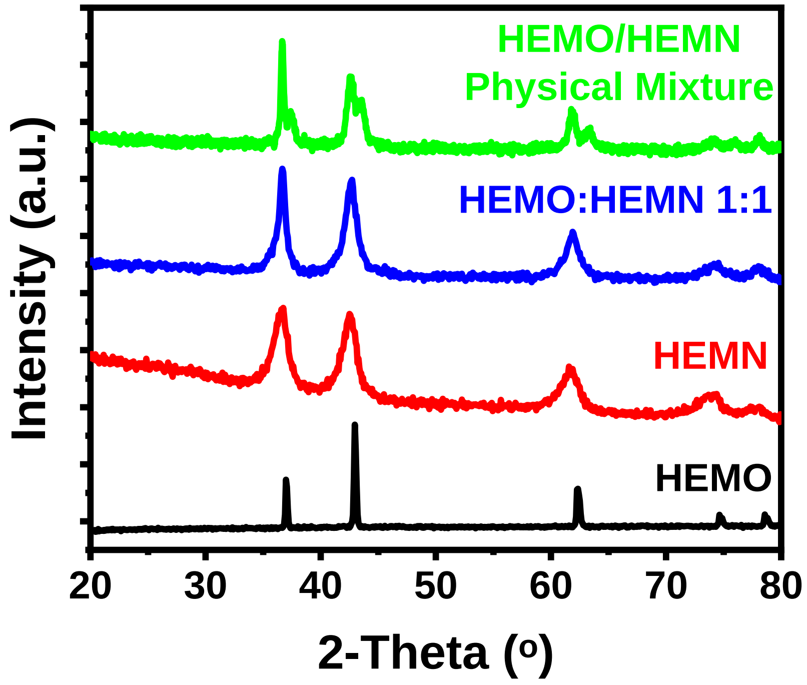

Figure S4. XRD Patterns of HEMO, HEMN, HEMO:HEMN 1:1, and physically mixed HEMO/HEMN.

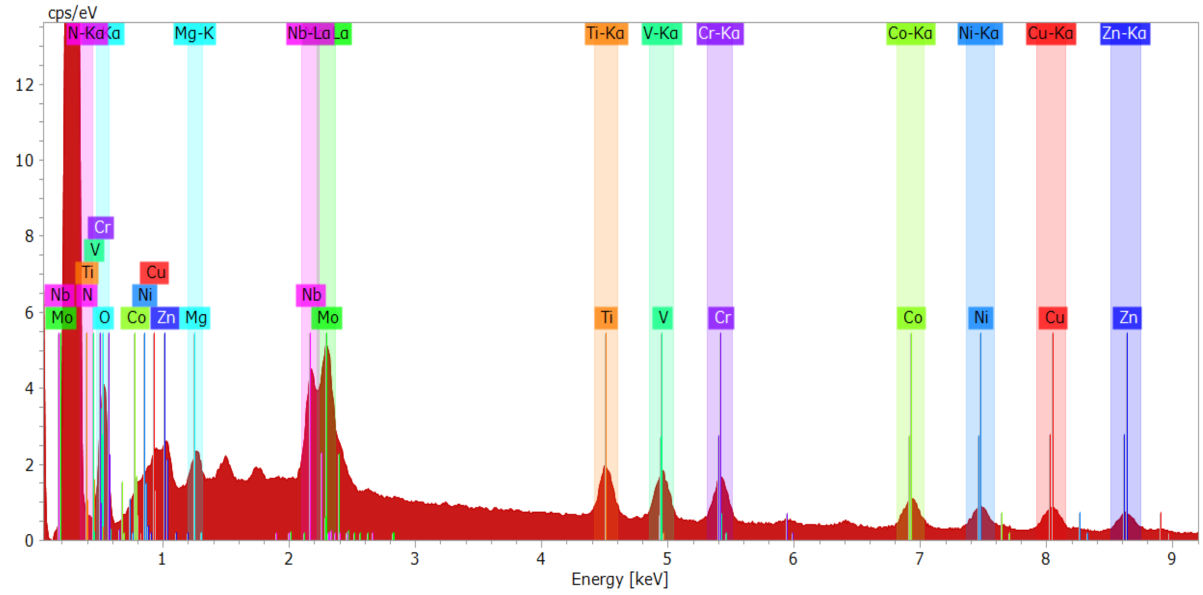

Figure S5. EDS Spectrum of HEMO:HEMN 1:1 featuring Zn, Ni, Co, Cu, Nb, V, Cr, N, Ti, Mo, Mg, O, and N signals.

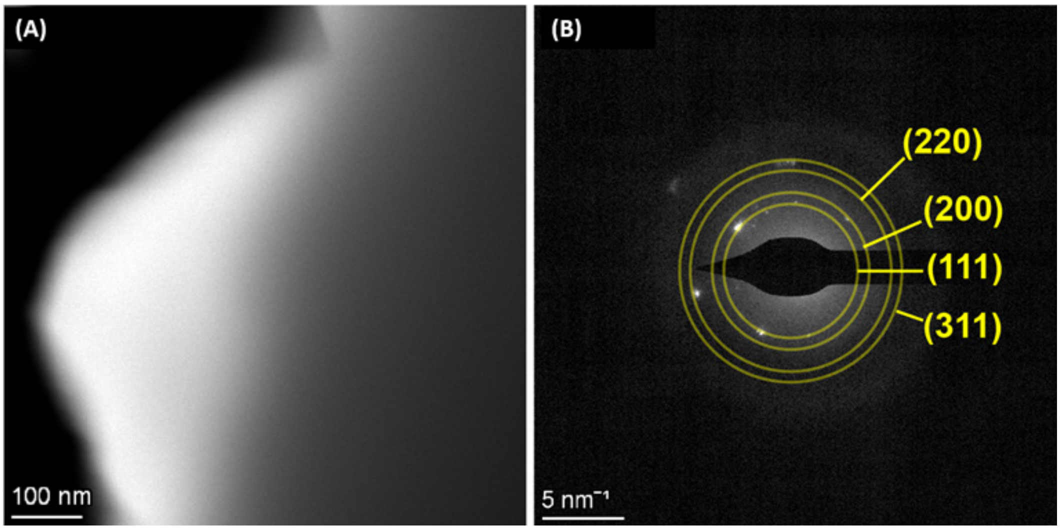

Figure S6. (A) HAADF-STEM and (B) SAED images of HEMO:HEMN 1:1 featuring (111), (200), (220), and (311) cubic phase diffraction rings.

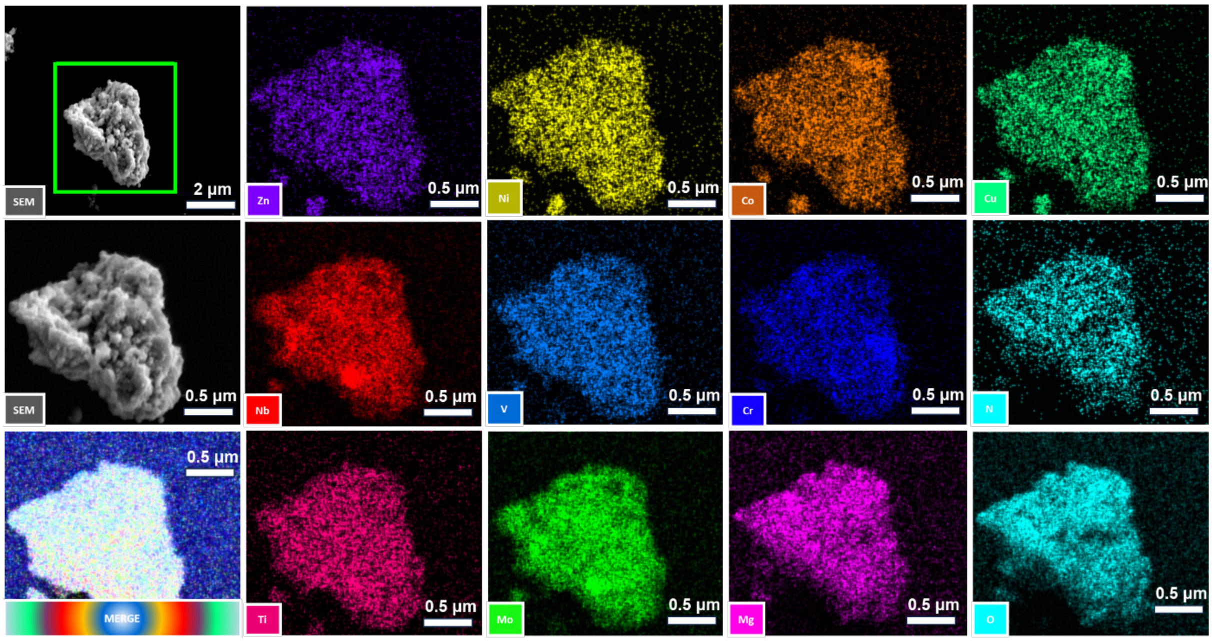

Figure S7. SEM and EDS mapping of HEMO:HEMN 2:1 featuring Zn, Ni, Co, Cu, Nb, V, Cr, N, Ti, Mo, Mg, and O signals.

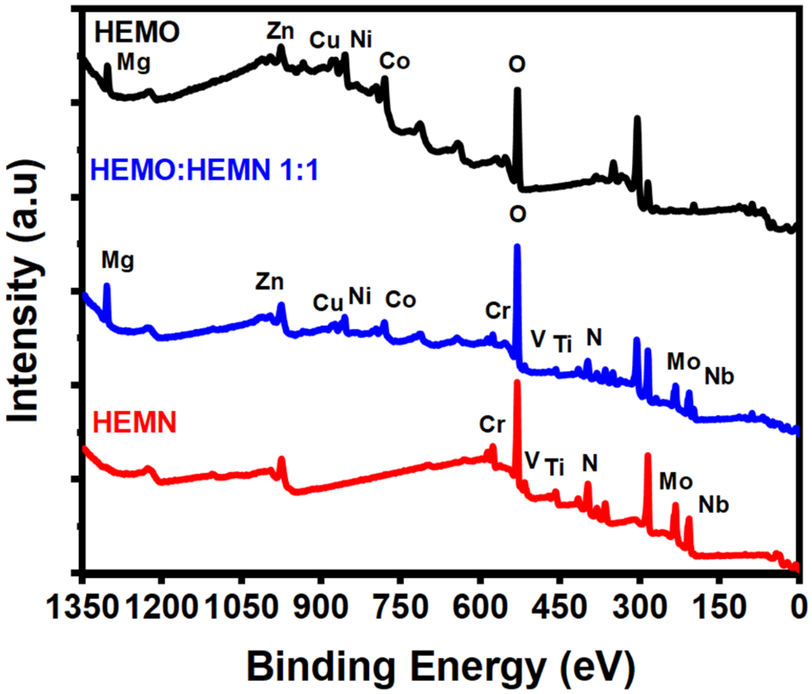

Figure S8. XPS survey spectra of HEMO, HEMN and HEMO:HEMN 1:1.

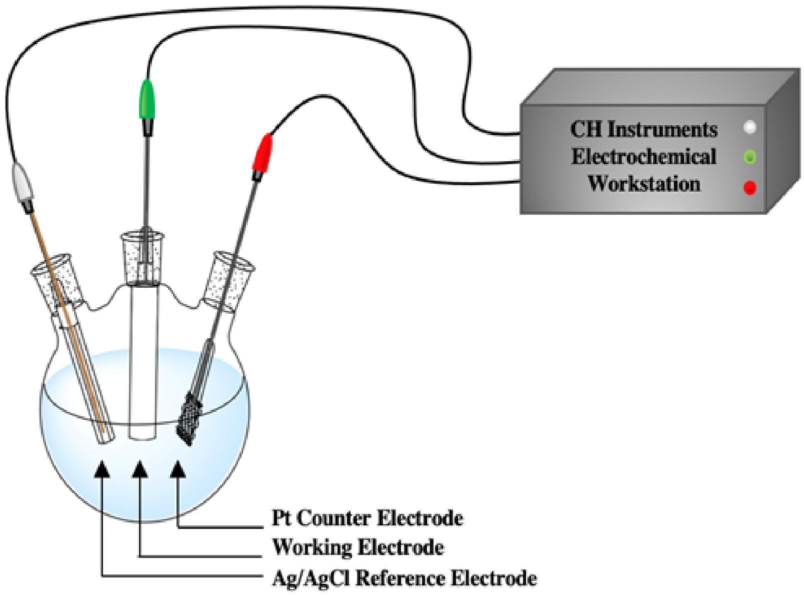

Figure S9. Schematic illustration of the three-cell electrode featuring a Platinum (Pt) counter electrode, glassy carbon working electrode, and Ag/AgCl reference electrode.

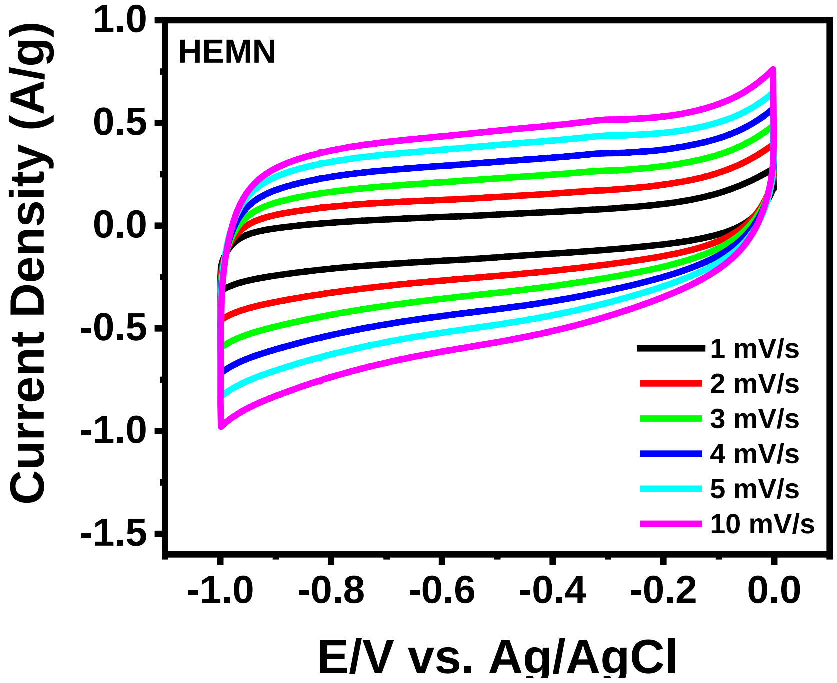

Figure S10. 1, 2, 3, 4, 5, and 10 mV s^{-1} scan rate CV curves for HEMN in 1M KOH.

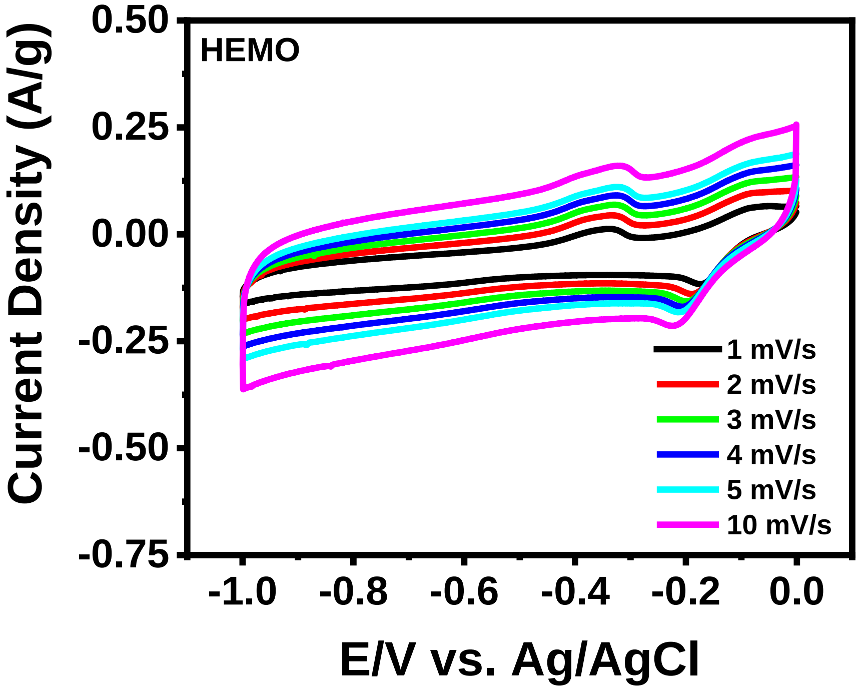

Figure S11. 1, 2, 3, 4, 5, and 10 mV s^{-1} scan rate CV curves for HEMO in 1M KOH.

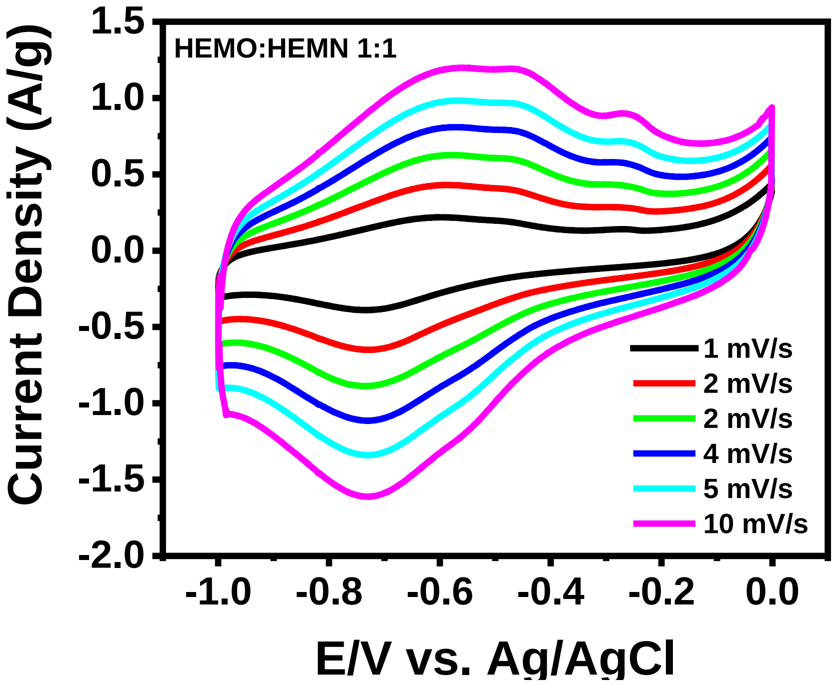

Figure S12. 1, 2, 3, 4, 5, and 10 mV s^{-1} scan rate CV curves for HEMO:HEMN 1:1 in 1M KOH.

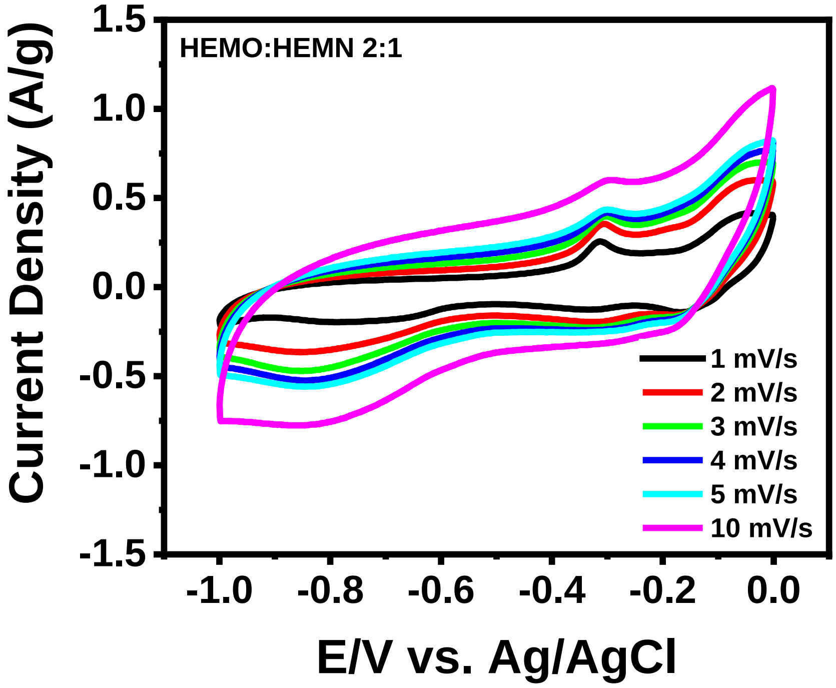

Figure S13. 1, 2, 3, 4, 5, and 10 mV s^{-1} scan rate CV curves for HEMO:HEMN 2:1 in 1M KOH.

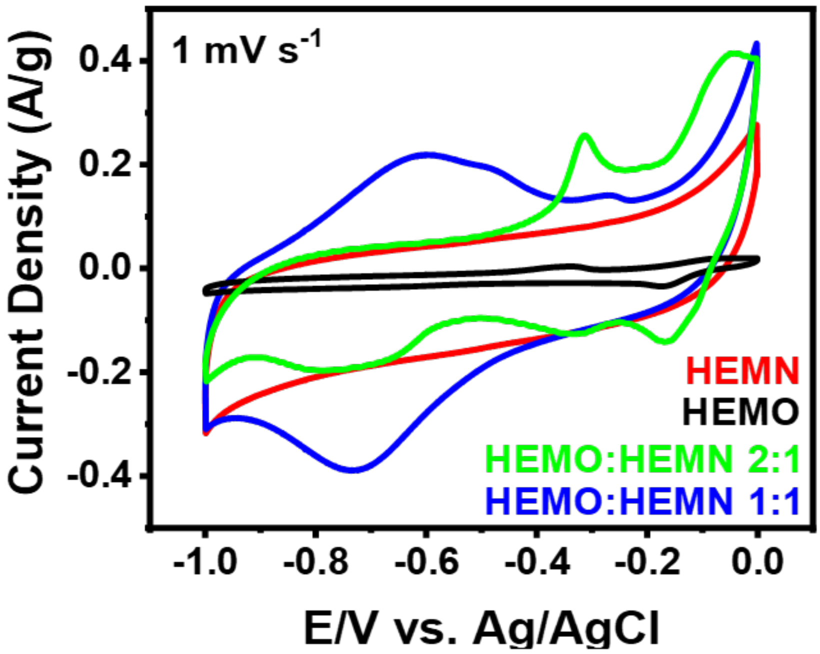

Figure S14. (A) CVs of HEMO, HEMN, HEMO:HEMN 1:1, and 2:1 in 1M KOH at a scan rate of 1 mV s-1.

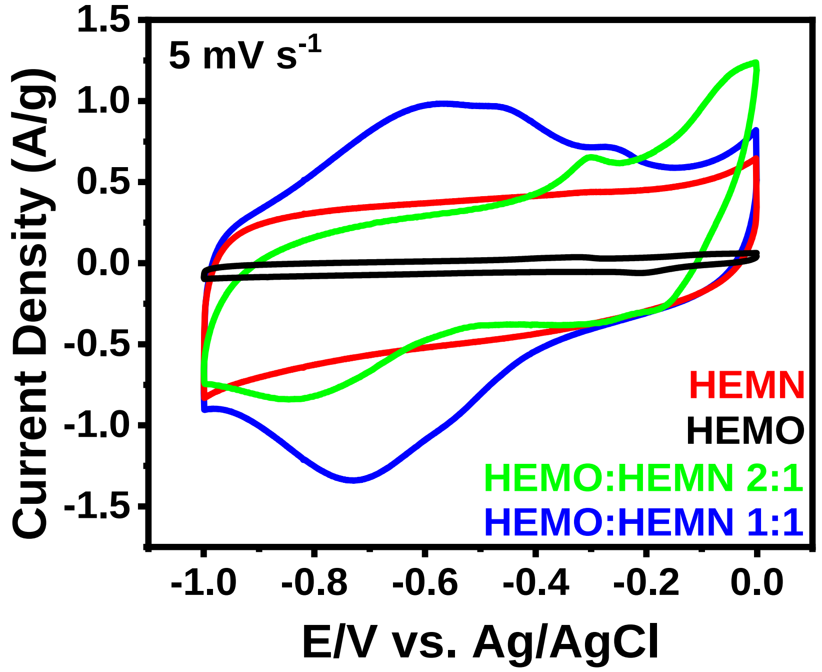

Figure S15. CV curves of HEMO, HEMN, HEMO:HEMN 1:1 and HEMO:HEMN 2:1 in 1M KOH at a scan rate of 5 mV s^{-1}.

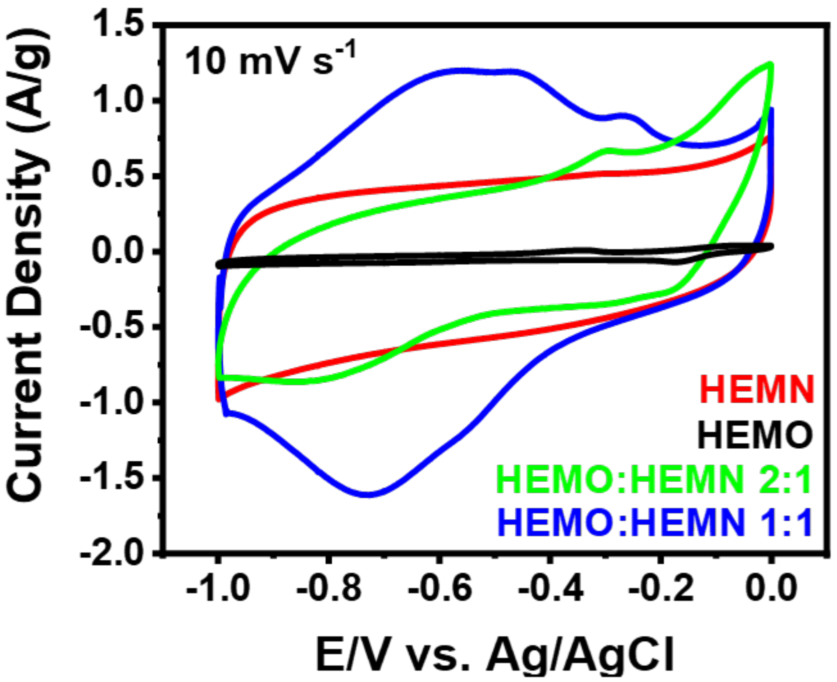

Figure S16. CV curves of HEMO, HEMN, HEMO:HEMN 1:1 and HEMO:HEMN 2:1 in 1M KOH at a scan rate of 5 mV s^{-1}.

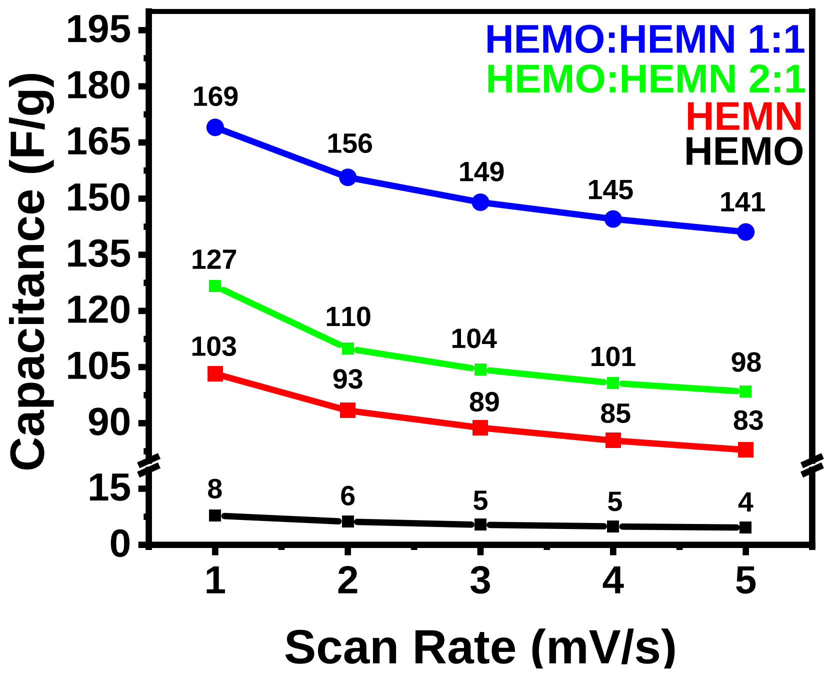

Figure S17. Capacitance performance of HEMO, HEMN, HEMO:HEMN 1:1, and 2:1 at scan rates 1-5 mV s-1.

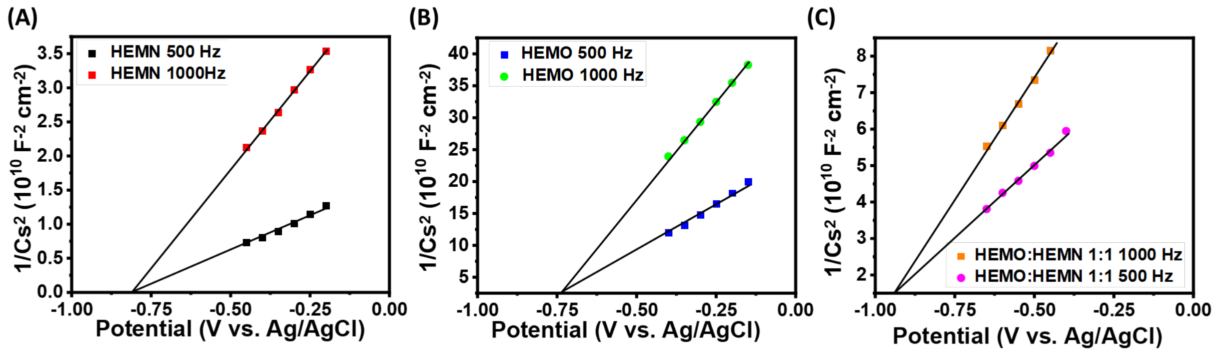

Figure S18. Mott Schottky Plots in the linear range of (A) HEMN, (B) HEMO, and (C) HEMO:HEMN 1:1 at 1000 Hz and 500 Hz.
